# miR-221-5p regulates proliferation and migration in human prostate cancer cells and reduces tumor growth in vivo

**DOI:** 10.1186/s12885-019-5819-6

**Published:** 2019-06-25

**Authors:** Mirjam Kiener, Lanpeng Chen, Markus Krebs, Joël Grosjean, Irena Klima, Charis Kalogirou, Hubertus Riedmiller, Burkhard Kneitz, George N. Thalmann, Ewa Snaar-Jagalska, Martin Spahn, Marianna Kruithof-de Julio, Eugenio Zoni

**Affiliations:** 1 Urology Research Laboratory, Department for BioMedical Research, University of Bern, Bern, Switzerland; 2 IBL, University of Leiden, Leiden, the Netherlands; 3 Department of Urology and Paediatric Urology, University Medical Center of Würzburg, Würzburg, Germany; 4 Department of Urology, Inselspital, Bern University Hospital, Bern, Switzerland; 5 Urology Center Zürich, Clinic Hirslanden, Zürich, Switzerland; 6 Department of Urology, Essen University Hospital, University of Duisburg-Essen, Essen, Germany

**Keywords:** Prostate cancer, miR-221-5p, Proliferation, Migration, Tumor suppressor miRNA

## Abstract

**Background:**

Despite latest advances in prostate cancer (PCa) therapy, PCa remains the third-leading cause of cancer-related death in European men. Dysregulation of microRNAs (miRNAs), small non-coding RNA molecules with gene expression regulatory function, has been reported in all types of epithelial and haematological cancers. In particular, miR-221-5p alterations have been reported in PCa.

**Methods:**

miRNA expression data was retrieved from a comprehensive publicly available dataset of 218 PCa patients (GSE21036) and miR-221-5p expression levels were analysed. The functional role of miR-221-5p was characterised in androgen- dependent and androgen- independent PCa cell line models (C4–2 and PC-3M-Pro4 cells) by miR-221-5p overexpression and knock-down experiments. The metastatic potential of highly aggressive PC-3M-Pro4 cells overexpressing miR-221-5p was determined by studying extravasation in a zebrafish model. Finally, the effect of miR-221-5p overexpression on the growth of PC-3M-Pro4luc2 cells in vivo was studied by orthotopic implantation in male Balb/cByJ nude mice and assessment of tumor growth.

**Results:**

Analysis of microRNA expression dataset for human primary and metastatic PCa samples and control normal adjacent benign prostate revealed miR-221-5p to be significantly downregulated in PCa compared to normal prostate tissue and in metastasis compared to primary PCa. Our in vitro data suggest that miR-221-5p overexpression reduced PCa cell proliferation and colony formation. Furthermore, miR-221-5p overexpression dramatically reduced migration of PCa cells, which was associated with differential expression of selected EMT markers. The functional changes of miR-221-5p overexpression were reversible by the loss of miR-221-5p levels, indicating that the tumor suppressive effects were specific to miR-221-5p. Additionally, miR-221-5p overexpression significantly reduced PC-3M-Pro4 cell extravasation and metastasis formation in a zebrafish model and decreased tumor burden in an orthotopic mouse model of PCa.

**Conclusions:**

Together these data strongly support a tumor suppressive role of miR-221-5p in the context of PCa and its potential as therapeutic target.

**Electronic supplementary material:**

The online version of this article (10.1186/s12885-019-5819-6) contains supplementary material, which is available to authorized users.

## Background

Prostate cancer (PCa) is the most commonly diagnosed cancer and the third leading cause of cancer-related death in European men [1]. Radical prostatectomy (RP) and radiotherapy are the standard of care to treat primary prostate adenocarcinoma and 70% of patients will be cured [2, 3]. Androgen deprivation therapy (ADT) is often applied as adjuvant treatment for radiotherapy or to treat advanced, hormone sensitive tumors [4] but most patients will develop castration-resistant PCa (CRPC) and progress to metastatic disease, which comprises the most lethal phase of PCa [2]. To date, treatment options for this stage of the disease are limited [5, 6]. New therapeutic strategies are therefore urgently required. miRNAs have been suggested as diagnostic and prognostic biomarkers and their application as new molecular therapeutic strategies in cancer and other diseases is currently under investigation [7–10].

MicroRNAs (miRNAs) are 19-25 nt long small non-coding RNAs that modulate a multitude of biological processes by negative regulation of their target mRNAs [11, 12]. miRNAs are encoded in introns or separate genes [13–15] and transcribed into a long primary transcript (pri-miR), which is cut by Drosha/DGCR8 complex into the ~ 70 nucleotide long precursor miRNA (pre-miRNA). In the cytoplasm, the pre-miRNA is processed by Dicer into the mature miRNA and incorporated into the Argonaut complex to regulate mRNA stability and translation via binding in the 3’UTR of its target mRNA [16, 17]. miRNAs have been found to be dysregulated in all types of cancer [18, 19] and some exert oncogenic or tumor suppressive function (oncomiRs) [20]. miR-221 is a cancer-associated miRNA [21]. It is encoded in a gene cluster together with miR-222 on the short arm of the X chromosome. miR-221/− 222 are transcribed together into the pri-miRNA, which is further processed to mature miR-221-3p and the passenger miR-221-5p to regulate gene expression [22].

miR-221 is overexpressed in a variety of epithelial cancers including breast, liver, bladder, pancreas, gastric, colorectal cancer, melanoma, papillary thyroid carcinoma and glioblastoma [21, 23]. In PCa miR-221 upregulation [24, 25] as well as miR-221 downregulation has been reported [26–30]. Due to the strong downregulation of miR-221 in PCa tissue and in the blood of PCa patients, miR-221 has been suggested as prognostic and predictive biomarker in PCa [31–33]. However, the role of miR-221 in tumorigenesis and cancer progression is controversial. miR-221-3p has been investigated extensively compared to miR-221-5p. In vivo studies demonstrated that miR-221/222 downregulation impairs the growth of PCa xenografts implying that miR-221-3p acts as oncogenic miRNA in PCa [34]. Moreover, miR-221-3p promotes proliferation of PCa cells by direct interaction and downregulation of p27/Kip1 [35]. Other tumor suppressors targeted by miR-221-3p include p57/Kip2, PTEN, TIMP3 and PUMA [36–38] . In contrast, miR-221-3p can act as tumor suppressor via downregulation of oncogenic c-kit, thereby regulating angiogenesis [39] and inhibiting the growth of erythroleukemic cells [40]. Moreover, it has been shown to reduce proliferation of PCa cells via downregulation of Bmi-1 [41], SOCS3 and IRF2 [42] and inhibit migration and invasion by targeting Ecm29 [28].

miR-221-5p exerts tumor suppressive function in colorectal cancer, reducing metastatic burden in vivo [43]. However, miR-221-5p shows oncogenic function by targeting SOCS1 in PCa cells. Consequently, silencing of miR-221-5p reduced tumor growth in vivo in a xenograft mouse model [44]. These studies indicate that despite relatively consistent reports of miR-221 downregulation in PCa its role in tumorigenesis is still unclear.

In this study we characterized the effects of miR-221-5p overexpression and downregulation in vitro in two human PCa cell lines (PC-3M-Pro4luc2 cells and C4–2 cells) and investigated the function of miR-221-5p in two in vivo models. Our results demonstrate tumor suppressive function of miR-221-5p in the context of PCa and support the inverse correlation between miR-221-5p expression and disease progression in microRNA expression dataset [45]. We show that miR-221-5p overexpression decreased proliferation and migration of PCa cell lines. Consistently with our in vitro data, miR-221-5p overexpression reduced tumor burden in vivo in a zebrafish model of cancer cell extravasation and in a xenograft mouse model of tumor growth.

## Methods

### Analysis of microRNA dataset

miR-221 (3p and 5p) expression in PCa patients was analysed in a publicly available dataset of 28 normal prostate samples and 113 tumor samples (99 primary and 14 metastatic samples; GSE21036) [45]. miR-221-5p and -3p expression data from GSE21036 dataset was extracted with R using shinyGEO [46].

### Cell culture

Normal prostatic epithelial cell line, Ep156T, was derived from normal tissue of a PCa patient undergoing radical prostatectomy [47] and cultured in MCDB-153 medium (Sigma Aldrich) containing 1.2 g/L NaHCO_3_ (Sigma Aldrich) and supplemented with 1% FBS (Seraglob, Bioswisstec Ltd), 1% Penicillin/Streptomycin (100 U/L/100 μg/ml; Sigma Aldrich), 1% non-essential amino acids (without L-Glutamine; Gibco®, ThermoFisher Scientific), 200 nM Hydrocortisone (Sigma Aldrich), 10 nM Dihydrotestosterone (DHT; Fluka Chemica), 10 nM Triiodothyronine (T3; Sigma Aldrich), 1% Insulin-transferrin-selenium (Gibco®, ThermoFisher Scientific), 5 ng/ml EGF (Peprotech), 50 μg/ml bovine pituitary extract (Gibco®, ThermoFisher Scientific) and 10 nM R1881 (LCG Group). PC-3M-Pro4luc2 cells originate from serial passage of PC-3M cells in the prostate of athymic mice [48] and were maintained in DMEM medium: DMEM containing 4.5 g/L D-Glucose and L-Glutamine (Gibco® LifeTechnologies™) supplemented with 10% FetalClone® II (HyClone™, GE Healthcare), 1% Penicillin/Streptomycin (100 U/L/100 μg/ml) and 0.8 mg/ml G418 Geneticin® (Gibco®, LifeTechnologies™) for the selection of luciferase positive clonal cell pools. The androgen-independent DU145 cell line was first isolated from a brain metastasis of a PCa patient [49] and was maintained in DMEM (4.5 g/L D-Glucose) medium supplemented with 10% FBS and 1% Penicillin/Streptomycin (100 U/L/100 μg/ml). VCaP cells [50] were derived from a bone metastasis of hormone refractory PCa and were maintained in RPMI 1640 medium with 2 g/L NaHCO_3_ and L-glutamine (Biochrom) supplemented with 10% FBS and 1% Penicillin/Streptomycin (100 U/L/100 μg/ml). LNCaP cells were originally derived from a lymph node metastasis [50] and grown in T-medium: DMEM containing 1 mg/L glucose (Sigma Aldrich) supplemented with 20% F12K (Gibco®, LifeTechnologies™), 10% FBS, 1% Insulin-transferrin-selenium, 13.6 pg/ml T3, 0.25μg/ml Biotin, 25 μg/ml Adenine and 1% Penicillin/Streptomycin (100 U/L/100 μg/ml). An androgen-independent cell line, C4–2 cells, was derived from LNCaP cells by passaging in castrated mice [50]. C4–2 cells were cultured in T-medium. All cell lines were maintained at 37 °C in an atmosphere containing 5% CO_2_ and were passaged when they reached a confluency of 70–90%. For the rescue experiment, cells were seeded and the total amount of cells was passaged in bigger cell culture flasks when they reached 80–90% confluency. The human cell lines employed for functional characterisation in this study have been authenticated using highly-polymorphic short tandem repeat loci (STRs).

### Cell cycle analysis

PC-3M-Pro4luc2 cells (20′000 cells/ml, 34′000 cells/ml and 40′000 cells/ml) and C4–2 cells (20′000 cells/ml, 34′000 cells/ml and 44′000 cells/ml) were seeded in 5 ml complete medium. The next day, a mild cell cycle arrest was induced by starvation for 48 h in the respective standard medium containing 0.3% FC II or 0.3% FBS, respectively. Starvation medium was replaced by complete medium containing 10% FC II or 10% FBS, respectively, and cell cycle progression was analysed at 0 h, 16 h and 48 h after cell cycle release. 250′000 cells were stained by Propidium iodide (PI) staining in Nicoletti buffer (0.1% Na_3_C_6_H_5_O_7_, 50 μg/ml PI and 0.1% Triton-X in water) at 4 °C for 30 min and DNA content measured by flow cytometry (BD™ LSR II). The percentage of cells in G1, S and G2/M phase was analysed in FlowJo™ 10 (FlowJo LLC) by applying the Dean-Jett-Fox model.

### Transfection

For transfection, 90′000 PC-3M-Pro4luc2 cells/ml and 150′000 C4–2 cells/ml were transfected with Lipofectamine® 2000 Reagent (Invitrogen) in 2 ml Opti-MEM® (Gibco®, LifeTechnologies™) according to the manufacturer’s protocol as described before [51]. Cells were transfected with 10 nM hsa-miR-221-5p pre-miR™ miRNA Precursor (miR-221-5p; assay ID: PM12613, Ambion), 10 nM Pre-miR™ Negative Control #1 (scrambled; assay ID: AM17110, Ambion), 10 nM Anti-miR™ miRNA Inhibitor hsa-miR-221-5p (anti-miR-221-5p; assay ID: AM12613, Ambion) or 10 nM Anti-miR™ miRNA Inhibitor Negative Control #1 (anti-scrambled; assay ID: AM17010, Ambion).

### MTS assay

Transfected PC-3M-Pro4luc2 cells and C4–2 cells were seeded at a density of 10′000 cells/ml or 20′000 cells/ml, respectively, in 150 μl complete medium. 20 μl 3-(4,5 dimethylthiazol-2-yl)-5-(3-carboxymethoxyphenyl)-2-(4- sulfophenyl)-2H-tetrazolium (MTS; CellTiter 96® AQueous One Solution Cell Proliferation Assay, Promega) were added to each well at indicated time points (0 h, 24 h, 48 h, and 72 h after seeding) and absorbance measured at 490 nm wavelength after 2 h incubation at 37 °C.

### Clonogenicity assay

PC-3M-pro4luc2 cells were seeded at a density of 50 cells/ml and C4–2 cells at 400 cells/ml in 2 ml complete DMEM or T-medium, respectively, and incubated at 37 °C for 14 days. The medium was re-freshed twice a week and after 2 weeks the cells were fixed in 4% paraformaldehyde (PFA) and stained with crystal violet (Merck) 1:20 diluted in distilled water. Pictures of plates were taken by ChemiDoc (BioRad) and colonies counted by ImageJ 1.51j8 [52].

### Migration assay

PC-3 M-Pro4luc2 cells and C4–2 cells were starved overnight in their respective standard medium containing 0.3% FC II or 0.3% FBS, respectively. The next day, 60′000 PC-3M-Pro4luc2 cells or 100′000 C4–2 cells were seeded in 500ul starvation medium in Boyden chambers with 8 μm pore size (Corning). Complete medium containing 10% FC II or 10% FBS, respectively, was added to the bottom of the well. PC-3M-Pro4luc2 cells and C4–2 cells were allowed to migrate for 22 h and 27 h, respectively, and then fixed in 4% PFA and stained with crystal violet as described by Zoni et al. [51]. Five images per chamber were taken by bright field microscope (Olympus) and migrated cells were counted.

### Zebrafish model

Tg(mpo:GFP)i114 zebrafish line [53, 54] was maintained according to standard protocols (www.ZFIN.org). PC-3 M-Pro4 cells fluorescently labelled with mCherry (PC-3M-Pro4_mCherry cells) were transfected with 10 nM miR-221-5p or 10 nM scrambled 48 h before inoculation. Zebrafish embryos were dechorionized at 2 days post fertilisation (dpf), anaesthetized with 0.003% tricaine (Sigma) and placed on 10 cm petri dish coated with 3% agarose. A single cell suspension of transfected PC-3M-Pro4_mCherry cells in PBS was kept at room temperature and was loaded into borosilicate glass capillary needles (1 mm O.D. × 0.78 mm I.D.; Harvard Apparatus). About 400 cells were injected above the ventral end of the Duct of Cuvier, where the Duct of Cuvier opens into the heart, using a Pneumatic Picopump and a manipulator (WPI). Data are representative of at least ≥20 embryos per group. Survival rate of control group lower than 80% was used as discard cut-off. After implantation, zebrafish embryos were maintained at 33 °C [55]. Images were taken at 1 day post injection (dpi) and 4dpi and red pixels were quantified by imageJ.

### Orthotopic mouse model

Male, immunocompromised Balb/cByJ nude mice were housed at the Central Animal Facility of the Medical Faculty of the University of Bern in a specific pathogen-free environment at a 12 h light/dark cycle. Mice had free access to autoclaved chow and water and were housed at a maximum of 5 animals/cage. Mice were maintained and checked daily according to animal license. Animal experiments were approved by the Canton of Bern, Switzerland (Permit Number: BE55/16), and carried out in accordance with the Swiss Guidelines for the Care and Use of Laboratory Animals and the ARRIVE guidelines. Mice were handled exclusively during day time at the Central Animal Facility of the Medical Faculty of the University of Bern. At the beginning of the experiment, mice were 8-weeks old and weighed 22 g ± 2 g. To conduct this study, a total of 10 mice was used to assess orthotopic growth of miR-221-5p overexpressing PC-3M-Pro4luc2 cells. PC-3M-Pro4luc2 cells were transfected with 10 nM miR-221-5p and 10 nM scrambled 48 h prior to inoculation. 8-weeks old male Balb/cByJ nude mice (Charles River France) were allocated to control group (scrambled: *n* = 5 mice) and experimental group (miR-221-5p = 5 mice). The mean body weight was comparable in both groups at beginning of the experiment. Mice were anaesthetized with Domitor® (0.5 mg/kg)/Dormicum® (5 mg/kg)/Fentanyl (0.05 mg/kg) triple narcosis mix subcutaneous (s.c.) injection, which is well tolerated by the mouse strain, and the eyes were protected by Vitamin A cream during surgery. Approximately 50′000 single cells were reconstituted in 10 μl PBS and injected into the right lobe of the anterior prostate. Surgeries were started in the morning with implantation of miR-221-5p transfected PC-3M-Pro4luc2 cells. Mice of the second cage (*n* = 5) were injected with scrambled transfected PC-3 M-Pro4luc2 cells. Immediately after surgery, the mice were s. c. injected with analgesic mix of Alzane® (1.1 mg/kg)/Anexate® (0.45 mg/kg)/Temgesic® (0.075 mg/kg) and received analgesia (Temgesic® 0.1 mg/kg) by s. c. injection twice a day for 3 days after surgery. Tumor growth was screened weekly by assessing bioluminescence with s. c. injection of 30 μl/mouse 250 mM D-Luciferin sodium salt (Synchem) and measuring by NightOWL II LB983 in vivo imaging system (Berthold Technologies). For BLI measurements, mice were anaesthetized by s. c. injection of Domitor® (0.5 mg/kg)/Dormicum® (5 mg/kg)/Fentanyl (0.05 mg/kg) triple narcosis mix and received an antidote mix of Alzane® (1.02 mg/kg)/Anexate® (0.42 mg/kg)/Naloxon (0.6 mg/kg) after measurement. Animals without signal along the course of the experiment were excluded from the analysis. At 28dpi the bioluminescence signal reached more than 10^6^cps and tumors were dissected. Tumor volume was calculated as π/6 x length x width^2^ [56] and normalised to the body weight (Additional file 1: Table S1). Tumor architecture and molecular markers were histologically characterised (see next paragraph). After tumor dissection, the experiment was terminated and all mice were euthanized by CO_2_ according to the recommendations of the Federal Veterinary Office.

### Histology and immunofluorescence stainings

Dissected tumors were formalin-fixed, paraffin-embedded (FFPE) and cut in 4 μm sections for staining by haematoxylin and eosin (H&E). Images were taken by Pannoramic 250 Flash II Scanner (3D Histech Ltd). For immunofluorescence, antigens of FFPE sections were retrieved in boiling citrate buffer and sections blocked in 1%BSA (Fluka)/PBS-Tween (Merck) for 30 min. The sections were stained for monoclonal anti-human proliferating cell nuclear antigen (PCNA; Cat.#: P8825, Sigma Aldrich) 1:500, polyclonal anti-human cleaved caspase-3 (cl. casp-3; Cat.#: 9661, Cell Signaling) 1:500, polyclonal anti-human pan-cytokeratin (panCK; Cat.#: A0575, DAKO) 1:250 and monoclonal anti-mouse α-smooth muscle actin (α-SMA; Cat.#: A2547, Sigma Aldrich) 1:400 in 1%BSA/PBS-Tween at 4 °C overnight. Subsequently, sections were incubated with secondary antibodies AlexaFluor 488 donkey anti-mouse (Cat.#: A-21202, LifeTechnologies™) 1:250 and AlexaFluor 555 donkey anti-rabbit (Cat.#: A-31572, LifeTechnologies™) 1:250 in PBS at room temperature for 1½ h. Images were taken by LCI Leica DMI4000 B microscope (Leica Microsystems).

For phalloidin staining, PC-3M-pro4luc2 cells transfected with 10 nM miR-221-5p or 10 nM scrambled were stained with Acti-stain 488 Phalloidin (Cat.#: 176753, abcam) according to manufacturer’s protocol. Briefly, 8′000 cells/well were seeded on 8 chamber polystyrene vessel tissue culture treated glass slides (Falcon) 24 h post transfection and let attach for 48 h. Cells were fixed in 4% PFA, permeabilised in 0.1% Triton-X in PBS and stained with Acti-stain 488 Phalloidin for 40 min at room temperature. Images were taken by LCI Leica DMI4000 B microscope and analysed by imageJ for mean phalloidin intensity normalised to the number of nuclei.

### RNA isolation and RT-qPCR

Total RNA was isolated from wild-type cell lines (Ep156T, LNCaP, C4–2, VCaP, PC-3M-Pro4luc2 and DU145 cells) or transfected PC-3M-pro4luc2 cells and C4–2 cells 72 h post transfection by TriPure Isolation Reagent (Roche) according to manufacturer’s protocol with an additional wash with 90% ethanol after RNA precipitation in isopropanol. For mRNA detection, cDNA was synthesised from 500 ng total RNA by M-MLV reverse transcriptase (Promega) and qPCR was performed by FastStart Universal SYBR Green Master Mix (Roche #04913850001) for EMT markers and housekeeping genes HPRT and β-actin (see Additional file 1: Table S2 for primer sequences). miRNA expression was analysed by cDNA synthesis from 2 ng total RNA by TaqMan™ Advanced miRNA cDNA Synthesis Kit (applied biosystems) and qPCR for hsa-miR-221-5p TaqMan™ Advanced miRNA Assays (assay ID: 478778_mir, applied biosystems) and housekeeping miRNAs hsa-miR-103a-3p TaqMan® Advanced miRNA Assays (assay ID: 478253_mir, applied biosystems) and hsa-miR-186-5p TaqMan® Advanced miRNA Assays (assay ID: 477940_mir, applied biosystems). Expression was normalised to housekeeping genes or miRNAs, respectively, and results are shown as relative expression calculated by 2^-ΔCt^ or as LOG difference calculated by LOG(2^-ΔΔCt^) method. Ct values > 35 were excluded from the analysis.

### Protein isolation and Western blot

Proteins were isolated 72 h post transfection with RIPA buffer (150 mM NaCl, 1% Triton-X, 1% sodium deoxycholate, 1% sodium dodecyl sulphate (SDS) in 25 mM Tris, pH 7.6) containing proteinase inhibitor (Roche) and phosphatase inhibitor (Roche). Protein was quantified by Pierce™ BCA protein assay kit (Thermo Scientific) according to manufacturer’s protocol for microplate procedure. 20 μg of protein were denatured in Lämmli buffer (BioRad) containing β-mercaptoethanol (Sigma Aldrich) by boiling at 95 °C for 10 min. Proteins were separated on a 12% SDS PAGE gel and transferred to a PVDF transfer membrane (Thermo Scientific). After blocking in 5% skimmed milk (Carl Roth GmbH + Co KG) in TBS-Tween the membranes were incubated with polyclonal goat anti-E-CAD (Cat.#: AF648, R&D Systems) 1:1000 or monoclonal mouse anti-VIM (Cat.#: ab8979, abcam) 1:1000 in 5% BSA in TBS-Tween at 4 °C overnight. The next day, membranes were incubated with mouse HRP-conjugated anti-β-actin (Cat.#: A3854, Sigma Aldrich) 1:20′000 in 5% skimmed milk and secondary antibodies HRP donkey anti-goat IgG (Cat.#: ab97110, abcam) 1:10′000 and ECL™ peroxidase labelled anti-mouse (Cat.#: NA931VS, GE Healthcare) 1:10′000 in TBS-Tween at room temperature for 1 h. WesternBright™ ECL-HRP substrate (Witec) was added to the membranes and images taken by Fusion-FX7–820 (Witec). Protein bands were quantified by imageJ as described by Luke Miller.org (https://lukemiller.org/index.php/2010/11/analyzing-gels-and-western-blots-with-image-j/).

### Statistics

The data was analysed by t-test, one-way ANOVA or two-way ANOVA as indicated in the figure legends with a confidence interval α ≤ 0.05. Data are shown as mean ± SD. *P* values smaller than 0.05 were considered as statistically significant (* *p* < 0.05, ** *p* < 0.01, *** *p* < 0.001, **** *p* < 0.0001). For BLI data of mouse experiment, normality plot of residuals was checked to make sure it approximately follows a straight line. All statistical tests were performed in GraphPad Prism 7.01 (GraphPad Software) or R (The R Foundation).

## Results

### miR-221-5p is downregulated in PCa and PCa progression

**Fig. 1:**
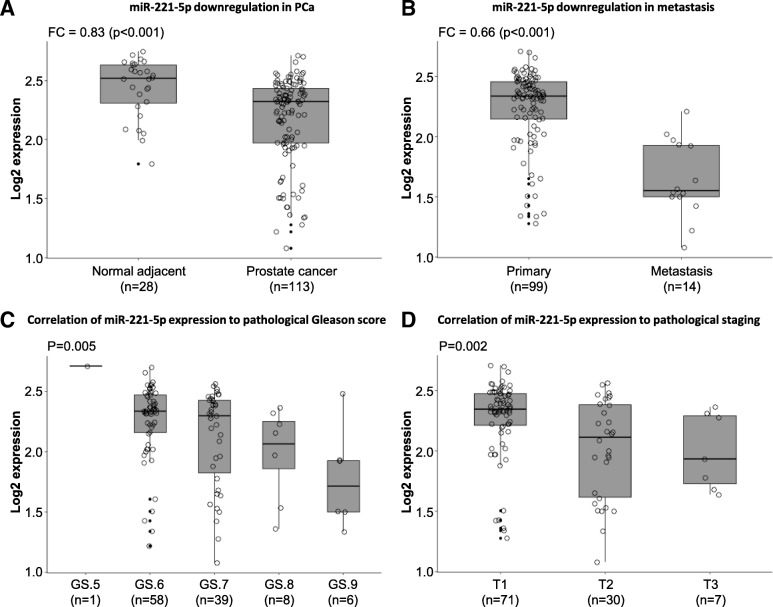
miR-221-5p is downregulated during PCa progression in patient samples. **a** Analysis of GSE21036 dataset [45] for miR-221-5p expression in PCa tissue compared to adjacent normal tissue. Fold change (FC = 0.83) to normal was calculated and data analysed by t-test. **b** Expression of miR-221-5p in 14 metastatic PCa samples was compared to miR-221-5p expression in 99 primary PCa tissue in GSE21036 dataset. Fold change (FC = 0.66) was calculated and data analysed by t-test. **c** Data of GSE21036 was grouped according to the indicated Gleason score (GS) and miR-221-5p expression analysed. Adjusted *p*-value was calculated by one-way ANOVA. **d** miR-221-5p expression was analysed in samples grouped for pathological stage (T). Data of GSE21036 was analysed by one-way ANOVA

To evaluate the relevance of miR-221 (5p and 3p) expression in PCa tissues, we analysed miR-221 expression in the Taylor dataset (GSE21036) [45] providing miRNA profiling data of tumor samples and normal adjacent tissue after radical prostatectomy. We observed significant downregulation of miR-221-5p (Fig. 1a; *p* < 0.001) and miR-221-3p (Additional file 2: Figure S1A; *p* < 0.001) in PCa samples compared to normal adjacent tissue. Further downregulation of miR-221-5p (Fig. 1b; *p* < 0.001) and miR-221-3p (Additional file 2: Figure S1B; *p* < 0.001) was detected in the progression to metastasis compared to primary tumor tissue. We furthermore analysed the correlation between miR-221 expression, Gleason score (GS) and tumor staging at surgery (RP) and diagnosis. Significant miR-221-5p and miR-221-3p downregulation was associated with increasing GS at surgery (Fig. 1c; *p* < 0.01 for miR-221-5p; Additional file 2: Figure S1C; *p* < 0.01 for miR-221-3p). In addition, miR-221-5p and miR-221-3p were downregulated with increasing pathological staging (Fig. 1d; *p* < 0.01 for miR-221-5p; Additional file 2: Figure S1D; *p* < 0.01 for miR-221-3p). However, we did not find a correlation between miR-221-5p or miR-221-3p expression and tumor staging at diagnosis (Additional file 2: Figure S1E & S1F). This study investigates specifically the functional role of miR-221-5p, given its less understood role in PCa compared to miR-221-3p.

### miR-221-5p overexpression reduces cell growth and colony formation in PCa cell lines

**Fig. 2:**
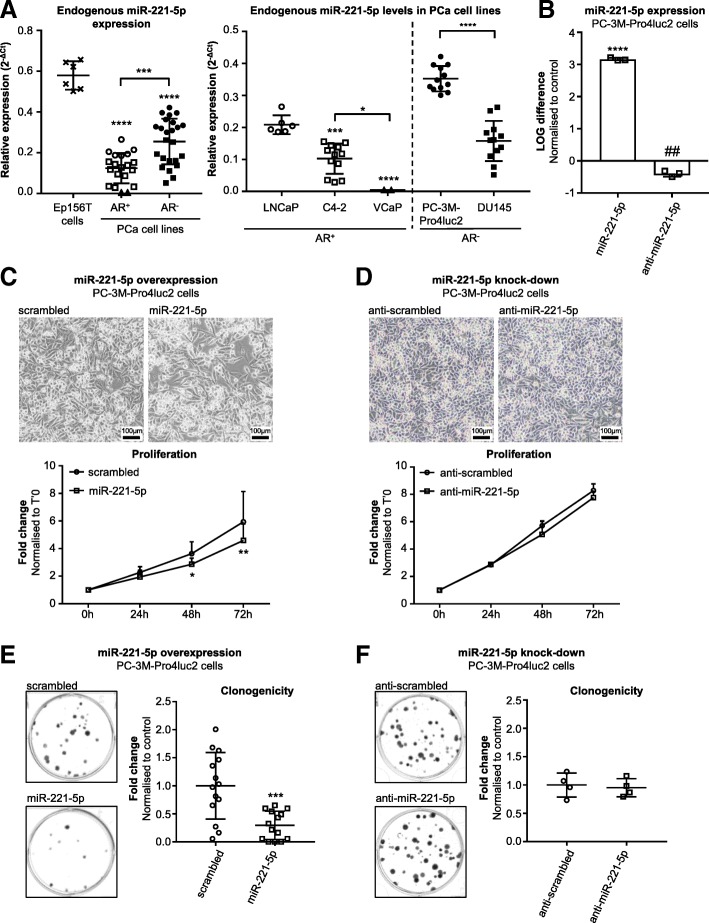
miR-221-5p exerts tumor suppressive function on PCa cell lines in vitro. **a** *Left*: Relative miR-221-5p expression (2^-ΔCt^) in normal prostatic epithelial Ep156T cells and AR^+^ and AR^−^ PCa cell lines. Analysis by one-way ANOVA with Tukey’s multiple comparisons test. *Right*: Technical replicates of at least two independent experiments for each cell line. miR-221-5p expression of AR^+^ cells was analysed by one-way ANOVA with Tukey’s multiple comparisons test. AR^−^ cell lines were compared by unpaired, two-tailed t-test. * *p* < 0.05, *** *p* < 0.001, **** *p* < 0.0001. Figure symbols: cross: Ep156T; circle: LNCaP; square: C4–2; triangle: VCaP; filled circle: PC-3M-Pro4luc2; filled square: DU145 cells. **b** miR-221-5p expression levels in PC-3M-Pro4luc2 cells upon overexpression or knock-down of miR-221-5p. LOG difference to control was calculated as LOG(2^-ΔΔCt^) and analysed by unpaired, two-tailed t-test. Three technical replicates of representative experiments are shown. **** *p* < 0.0001 vs. scrambled, ## *p* < 0.01 vs. anti-scrambled. **c** Effect of miR-221-5p on proliferation. Images were taken 72 h post transfection with miR-221-5p and scrambled. MTS assay at 0 h, 24 h, 48 h, 72 h. Fold change compared to time point T’0 h was calculated for each time point. Data of *n* = 4 independent experiments are shown and were analysed by two-way ANOVA repeated measures by both factors with Sidak’s multiple comparison test. * *p* < 0.05, ** *p* < 0.01. **d** Effect of anti-miR-221-5p on proliferation. Images were taken at 72 h post transfection. MTS assay at 0 h, 24 h, 48 h, 72 h. Fold change compared to time point T’0 h was calculated for each time point. Data represent *n* = 5 technical replicates and were analysed by two-way ANOVA repeated measures with Sidak’s multiple comparison test. **e** Clonogenicity assay in miR-221-5p overexpressing PC-3M-Pro4luc2 cells. Dots indicate technical replicates of *n* = 4 independent experiments. Fold change was calculated vs. scrambled control and analysed by unpaired, two-tailed t-test. *** *p* < 0.001. **f** Clonogenicity assay in miR-221-5p knocked-down PC-3M-Pro4luc2 cells. *N* = 4 technical replicates are shown as fold change to control and analysed by unpaired, two-tailed t-test

Endogenous miR-221-5p expression levels were studied by Advanced miR TaqMan RT-qPCR in the normal prostatic epithelial cell line Ep156T and different PCa cell lines. We selected androgen receptor positive (AR^+^) LNCaP cells, C4–2 cells and VCaP cells and androgen receptor negative (AR^−^) PC-3M-Pro4luc2 and DU145 cell lines. The highest miR-221-5p expression was observed in normal prostatic epithelial Ep156T cells compared to PCa cell lines, regardless of AR status (Fig. 2a left panel; *p* < 0.0001). Interestingly, AR^−^ PCa cells expressed significantly higher miR-221-5p levels than AR^+^ PCa cell lines (Fig. 2a left panel; *p* < 0.001). Notably, miR-221-5p was differentially expressed among AR^+^ cell lines (Fig. 2a right panel). Androgen-sensitive LNCaP cells expressed higher miR-221-5p levels than the LNCaP-derived, androgen-independent C4–2 cell line (*p* < 0.001). Most pronounced downregulation of miR-221-5p was observed in VCaP cells, a cell line derived from human bone metastatic tissue (*p* < 0.0001 compared to LNCaP cells, *p* < 0.05 compared to C4–2 cells). The highest miR-221-5p expression was observed in PC-3M-Pro4luc2 cells, which was significantly higher than in DU145 cells (Fig. 2a right panel; *p* < 0.0001).

The AR^−^ PC-3 M-Pro4luc2 cells displaying high levels of miR-221-5p and AR^+^ C4–2 cells expressing low endogenous miR-221-5p levels were chosen to further characterise miR-221-5p function in vitro. Despite higher miR-221-5p expression, PC-3 M-Pro4luc2 cells proliferated faster than C4–2 cells. Cell cycle analysis by PI staining showed a significantly higher fraction of PC-3 M-Pro4luc2 cells proceeding from G1 to S-phase at 16 h after release from cell cycle arrest resulting in an increased fraction of PC-3M-Pro4luc2 cells in G2/M phase compared to C4–2 cells at 48 h (Additional file 3: Figure S2A; *p* < 0.01 for G1 and S-phase at 16 h; *p* < 0.05 for G2/M at 48 h). The effect of miR-221-5p on PC-3M-Pro4luc2 and C4–2 cells was further studied by overexpression and knock-down experiments. PC-3M-Pro4luc2 cells were transfected with 10 nM miR-221-5p or 10 nM anti-miR-221-5p in order to overexpress or knock-down miR-221-5p (Fig. 2b; *p* < 0.0001 for overexpression; *p* < 0.01 for knock-down; data normalized to respective scrambled controls). Specificity of the anti-miR-221-5p was confirmed by a co-transfection experiment. Anti-miR-221-5p significantly antagonized the miR-221-5p overexpression at concentrations as low as 10 nM (Additional file 3: Figure S2B; *p* < 0.0001). At 120 nM concentration, anti-miR-221-5p partially rescued miR-221-5p overexpression (Additional file 3: Figure S2B; *p* < 0.0001). Similar miR-221-5p induction upon transfection of miR-221-5p was achieved in the C4–2 cells (Additional file 3: Figure S2C; *p* < 0.0001) and we assessed cell proliferation of miR-221-5p transfected cells. miR-221-5p overexpression significantly decreased proliferation in PC-3M-Pro4luc2 cells (Fig. 2c; *p* < 0.05 at 48 h, *p* < 0.01 at 72 h) and C4–2 cells (Additional file 3: Figure S2D; *p* < 0.001 at 48 h, *p* < 0.0001 at 72 h) compared to control. miR-221-5p knock-down in PC-3M-Pro4luc2 cells resulted in similar proliferation rate compared to control (Fig. 2d).

The ability for unlimited self-renewal was studied by clonogenicity assay. Overexpression of miR-221-5p strongly reduced the number of colonies in PC-3M-Pro4luc2 cells (Fig. 2e; *p* < 0.001) and C4–2 cells (Additional file 3: Figure S2E; *p* < 0.0001) whereas this was not altered by miR-221-5p knock-down (Fig. 2f). These data suggest a role of miR-221-5p in PCa cell proliferation.

### miR-221-5p affects cell plasticity and reduces cell migration in vitro and extravasation in vivo

**Fig. 3:**
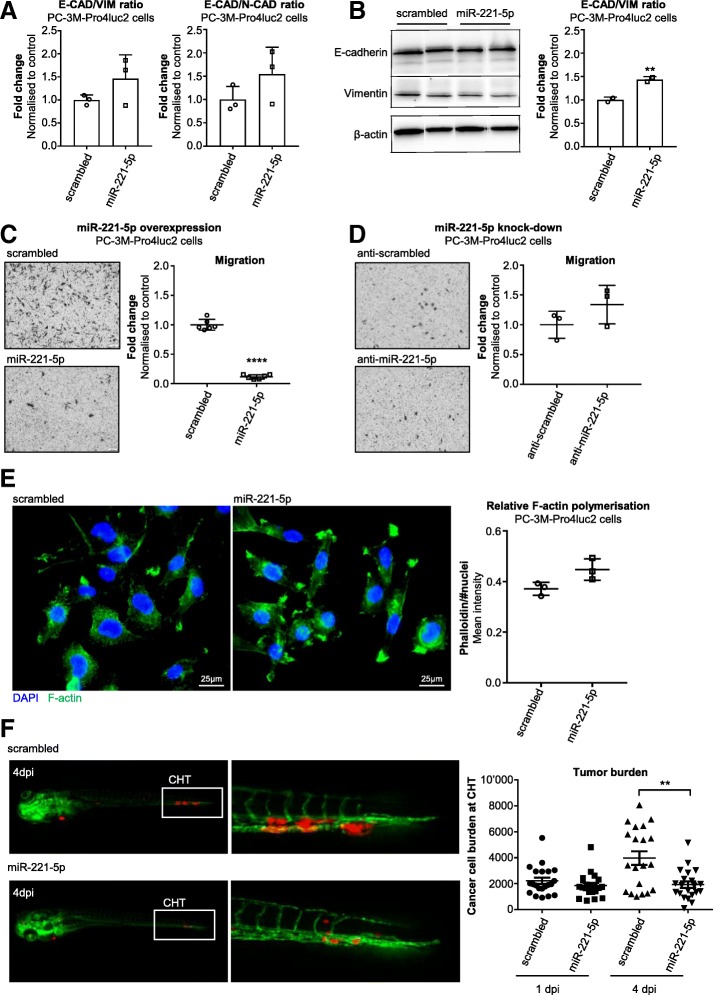
Overexpression of miR-221-5p affects plasticity of PCa cells and reduces extravasation in vivo*.* **a** E-CAD/N-CAD and E-CAD/VIM ratios were calculated from the relative mRNA expression and data of *n* = 3 independent experiments are shown as fold change to control. Data were analysed by paired, two-tailed t-test. **b** Expression of E-CAD and VIM protein in PC-3M-Pro4luc2 cells 72 h post transfection with miR-221-5p and scrambled. Bands were quantified and E-CAD/VIM ratio was calculated (normalised to β-actin). The data of *n* = 2 independent experiments are shown as fold change to scrambled and were analysed by paired, two-tailed t-test. ** *p* < 0.01. **c** Migration of miR-221-5p overexpressing PC-3M-Pro4luc2 cells. Dots represent technical replicates of *n* = 3 independent experiments. Data are shown as fold change to scrambled and were analysed by unpaired, two-tailed t-test. **** *p* < 0.0001. **d** Migration of PC-3M-Pro4luc2 cells with miR-221-5p knock-down. *N* = 3 technical replicates are shown. Data were normalised to control and analysed by unpaired, two-tailed t-test. **e** Phalloidin staining in PC-3M-Pro4luc2 cells overexpressing miR-221-5p or scrambled control. F-actin signal was normalised to number of nuclei. Data was analysed by unpaired, two-tailed t-test. F-actin = green; DAPI = blue. **f** Extravasation of miR-221-5p and scrambled transfected PC-3M-pro4_mCherry cells at the caudal hematopoietic tissue (CHT) of zebrafish embryos 4 days post injection (dpi). Tumor burden was quantified 1dpi and 4dpi. Green = vessels; red = PC-3M-pro4_mCherry cells. Data was analysed by unpaired, two-tailed t-test. ** *p* < 0.01

To understand whether miR-221-5p overexpression induces a more epithelial phenotype, RNA was isolated from miR-221-5p overexpressing PC-3M-Pro4luc2 cells and C4–2 cells 72 h post transfection. The expression of selected EMT markers including E-cadherin (E-CAD), N-cadherin (N-CAD), vimentin (VIM), zinc finger E-box-binding homeobox 2 (ZEB-2), SNAIL-1, SNAIL-2 and TWIST was analysed (Additional file 4: Figure S3A & S3B). Subsequently, E-CAD/N-CAD and E-CAD/VIM mRNA ratios were calculated. E-CAD mRNA expression was significantly increased by miR-221-5p overexpression in PC-3M-Pro4luc2 cells (Additional file 4: Figure S3A; *p* < 0.05) leading to an increase in the E-CAD/N-CAD and E-CAD/VIM mRNA ratio although this did not reach significance (Fig. 3a). Notably, in C4–2 cells VIM was significantly decreased by miR-221-5p overexpression (Additional file 4: Figure S3B; *p* < 0.05). As a consequence, E-CAD/VIM mRNA ratio was significantly increased in miR-221-5p transfected C4–2 cells (Additional file 4: Figure S3C; *p* < 0.05). We measured the effect of miR-221-5p on E-CAD and VIM protein by western blot. E-CAD expression remained unchanged while VIM showed a trend for downregulation by miR-221-5p overexpression compared to control in PC-3M-Pro4luc2 cells (Fig. 3b and Additional file 4: Figure S3D). The E-CAD/VIM protein ratio was significantly elevated in miR-221-5p transfected PC-3M-Pro4luc2 cells compared to control (Fig. 3b; *p* < 0.01). In C4–2 cells, we observed a trend for E-CAD protein downregulation upon miR-221-5p overexpression (Additional file 4: Figure S3E & S3F) resulting in decreased E-CAD/VIM ratio (Additional file 4: Figure S3F; *p* < 0.05).

To evaluate whether the differential expression of EMT markers in miR-221-5p transfected cells correlates with functional changes, we studied the effect of miR-221-5p on migration by transwell migration assay. The migration of PC-3M-Pro4luc2 cells was significantly reduced by miR-221-5p overexpression (Fig. 3c; *p* < 0.0001) while miR-221-5p knock-down had no effect on migration (Fig. 3d). Consistently, miR-221-5p overexpression resulted also in decreased migration in C4–2 cells (Additional file 4: Figure S3G; *p* < 0.0001).

Moreover, we investigated if miR-221-5p affected the structure of the cytoskeleton by phalloidin staining on miR-221-5p transfected PC-3M-Pro4luc2 cells. F-actin polymerisation was increased, though not significant, by miR-221-5p transfection in PC-3M-Pro4luc2 cells (Fig. 3e). We did not observe a difference in the morphology of the cells.

We studied the effect of miR-221-5p on extravasation in vivo in our established zebrafish model [51, 57]. Fluorescently labelled, transiently miR-221-5p overexpressing PC-3M-Pro4 cells expressing the fluorescent protein mCherry (PC-3M-Pro4_mCherry) were injected into the Duct of Cuvier of zebrafish embryos 2 days post fertilization (dpf). PC-3M-Pro4_mCherry cells overexpressing miR-221-5p formed fewer and smaller metastatic foci in the caudal hematopoietic tissue (CHT) and the tumor burden was reduced by miR-221-5p overexpression 4 days post injection (dpi) (Fig. 3f; *p* < 0.01).

Taken together, these data suggest that miR-221-5p overexpression modulates EMT markers and migration in vitro. In line with our in vitro data, miR-221-5p overexpression decreases extravasation at CHT in zebrafish leading to reduced metastatic growth.

### miR-221-5p overexpression reduces prostate tumor growth in vivo

**Fig. 4:**
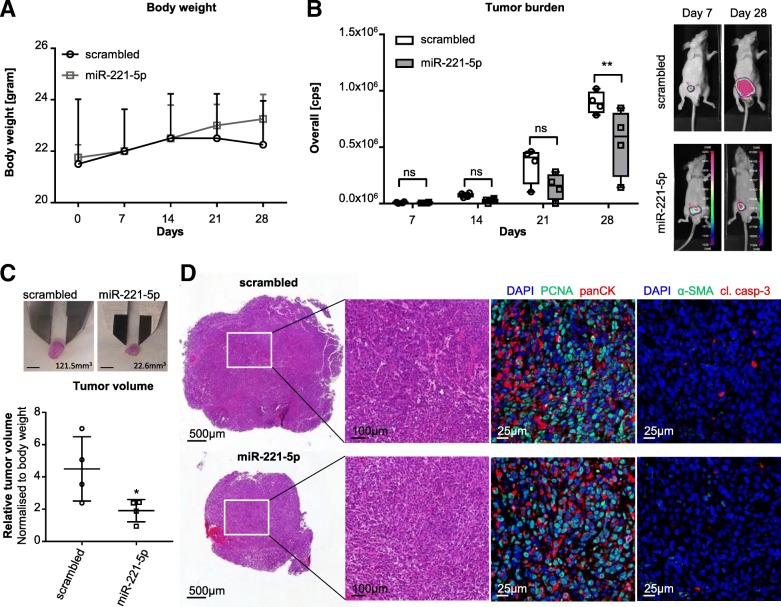
Orthotopic tumor growth is reduced by miR-221-5p overexpression in vivo*.* **a** Body weight was measured weekly along the course of the in vivo experiment (*n* = 4 for miR-221-5p and scrambled control). Data was analysed by two-way ANOVA repeated measures with Sidak’s multiple comparison test. **b** BLI imaging of mice injected with miR-221-5p or scrambled negative control. One representative image of each group is shown at 7 and 28 days. Tumor growth was quantified by overall counts per second (cps) from four animals per group (*n* = 4). The data was analysed by two-way ANOVA repeated measures with Sidak’s multiple comparison test. ** *p* < 0.01. **c** Tumor volume of miR-221-5p and scrambled overexpressing PC-3 M-Pro4luc2 cells was calculated as V = π/6 x length x width^2^ and normalised to the body weight. Data was analysed by unpaired, two-tailed t-test. * *p* < 0.05. Scale bar = 5 mm. **d** H&E of scrambled and miR-221-5p group (left panel). The expression of proliferation marker PCNA (green; middle panel), apoptosis marker cleaved caspase-3 (cl. casp-3; red; right panel), tumor cell marker panCK (red; middle panel) and stroma marker α-SMA (green; right panel) was analysed by immunofluorescence staining

To investigate the effect of miR-221-5p on tumor growth, we studied miR-221-5p overexpressing PC-3M-Pro4luc2 cells in vivo in an orthotopic mouse model. PC-3M-Pro4luc2 cells were transfected with miR-221-5p or scrambled and injected into the anterior prostate of Balb/cByJ nude male mice. Overexpression of miR-221-5p was confirmed by RT-qPCR (Additional file 5: Figure S4A; *p* < 0.0001) and clonogenicity was tested on the same batch of cells used for anterior prostatic implantation (Additional file 5: Figure S4B). Tumor growth was screened weekly by bioluminescence imaging (BLI) (Additional file 5: Figure S4C) and body weight was registered along the course of the experiment (Fig. 4a). miR-221-5p overexpression significantly reduced tumor growth compared to control in vivo (Fig. 4b; *p* < 0.01, 28 days after cells implantation). Moreover, miR-221-5p overexpression resulted in a significantly lower tumor volume (normalised to body weight) compared to control group (Fig. 4c; *p* < 0.05; Additional file 5: Figure S4D and Additional file 1: Table S1). Histological evaluation by hematoxilin and eosin (H&E) staining revealed no difference between miR-221-5p overexpressing and control tumors (Fig. 4d and Additional file 6: Figure S5). The sections were stained for proliferation and apoptosis markers (proliferating cell nuclear antigen (PCNA) and cleaved caspase-3 (cl. casp-3)). Despite the differences in tumor growth and volume, no difference in the level of proliferating and apoptotic cells between miR-221-5p and control was detected by PCNA and cl. casp-3 immunofluorescence staining (Fig. 4d and Additional file 6: Figure S5). In order to evaluate whether miR-221-5p levels affected the tumor composition regarding tumor cell burden and stroma contribution, the tumor sections were stained for tumor cell specific marker panCK and stromal marker α-smooth muscle actin (α-SMA). We observed a similar pattern between miR-221-5p and scrambled control (Fig. 4d and Additional file 6: Figure S5).

Taken together, these data demonstrated that miR-221-5p overexpression reduced tumor growth in vivo without affecting apoptosis or the features of the tumor associated stroma.

### Loss of miR-221-5p overexpression rescues proliferation, colony formation and migration capacity of aggressive PCa cells in vitro

**Fig. 5:**
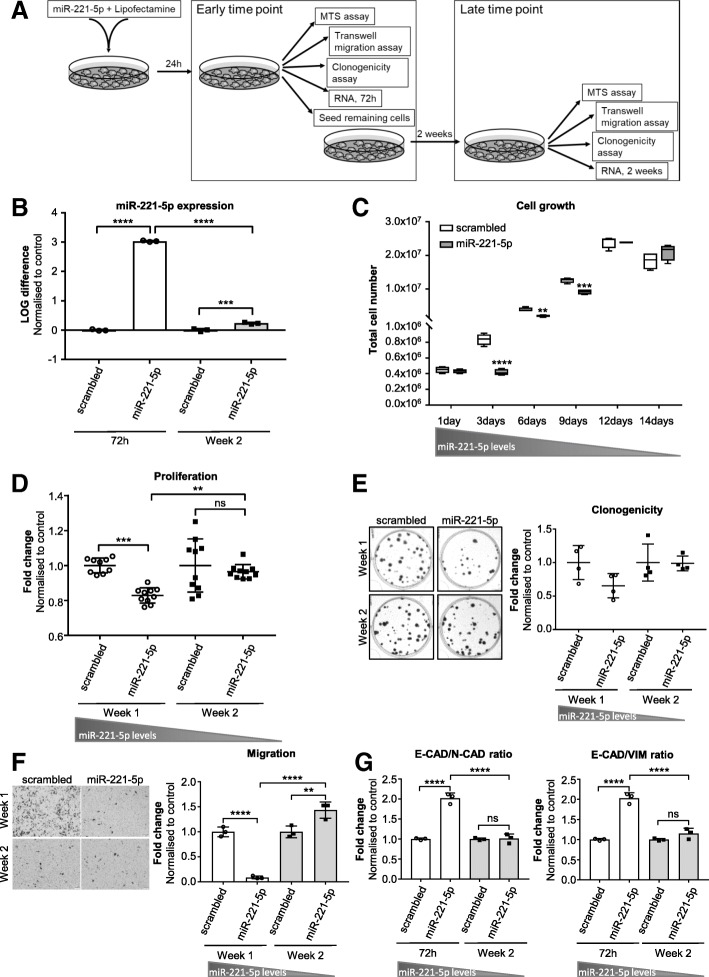
miR-221-5p-induced functional changes correlate to miR-221-5p (over)expression levels. **a** Experimental design of the rescue experiment after miR-221-5p overexpression. **b** miR-221-5p expression in PC-3M-Pro4luc2 cells 72 h and 2 weeks post transfection. miR-221-5p expression was normalised to housekeeping miRNAs and LOG difference to control (LOG(2^-ΔΔCt^)) is shown. Data are representative of *n* = 3 technical replicates and were analysed by one-way ANOVA with Tukey’s multiple comparison. *** *p* < 0.001, **** *p* < 0.0001. **c** Cell growth estimated by counting the total number of cells at different time points post transfection (1, 3, 6, 9, 12 and 14 days). Data represent technical replicates and were analysed by two-way ANOVA repeated measures with Sidak’s multiple comparison test. ** *p* < 0.01, *** *p* < 0.001, **** *p* < 0.0001. **d** MTS assay for proliferation of miR-221-5p or scrambled transfected PC-3M-Pro4luc2 cells starting 24 h or 2 weeks post transfection. Data of *n* = 10 technical replicates were normalised to T’0 h and fold change to scrambled at T’72 h was calculated. Data were analysed by one-way ANOVA with Tukey’s multiple comparison test. ** *p* < 0.01, *** *p* < 0.001. **e** Clonogenicity assay in PC-3M-Pro4luc2 cells overexpressing miR-221-5p or scrambled control at early and late time point. Data are representative of *n* = 4 technical replicates and fold change vs. scrambled represented. Data was analysed by one-way ANOVA with Tukey’s multiple comparison test. **f** Migration assay in PC-3M-Pro4luc2 cells overexpressing miR-221-5p or scrambled negative control at 1 and 2 weeks post transfection. The number of migrated cells was counted and the fold change to scrambled calculated. Data of technical triplicates was analysed by one-way ANOVA with Tukey’s multiple comparison test. ** *p* < 0.01, **** *p* < 0.0001. **g** E-CAD/N-CAD and E-CAD/VIM mRNA ratio of miR-221-5p and scrambled overexpressing PC-3M-Pro4luc2 cells. Data of *n* = 3 technical replicates are shown as fold change to scrambled and were analysed by one-way ANOVA with Tukey’s multiple comparison test. **** *p* < 0.0001

To investigate the kinetic of miR-221-5p expression following transfection, we evaluated miR-221-5p expression levels at different time points, early after transfection (72 h post transfection) and at 2 weeks. miR-221-5p expression levels were correlated to functional changes by assessing proliferation, clonogenicity and migration at an early time point and a late time point (Fig. 5a). RNA was isolated from transfected cells 72 h and 2 weeks post transfection (Fig. 5a). miR-221-5p was significantly overexpressed 72 h post transfection compared to scrambled control (Fig. 5b; *p* < 0.0001 at 72 h). After 2 weeks in culture, the miR-221-5p levels had significantly decreased (*p* < 0.0001) though were still significantly overexpressed compared to control (Fig. 5b; *p* < 0.001 at week 2). Overexpression of miR-221-5p led to significantly reduced cell number than control at day 3 (*p* < 0.0001), day 6 (*p* < 0.01) and day 9 (*p* < 0.001) post transfection suggesting reduced cell growth (Fig. 5c). At day 12 post transfection, the cell number in miR-221-5p transfected cells equalled to control (Fig. 5c). At the early time point, proliferation (Fig. 5d; *p* < 0.001 at week 1), clonogenicity (Fig. 5e) and migration (Fig. 5f; *p* < 0.0001 at week 1) of miR-221-5p transfected PC-3M-Pro4luc2 cells were reduced compared to control though clonogenicity did not reach significance. At the late time point, the effect of miR-221-5p overexpression on proliferation (Fig. 5d; *p* < 0.01), clonogenicity (Fig. 5e) and migration (Fig. 5f; *p* < 0.0001) was lost compared to early time point. Notably, the migration capacity of miR-221-5p transfected cells was significantly higher compared to control at the late time point (Fig. 5f; *p* < 0.01 at week 2). We also evaluated the expression of selected EMT markers at the early and late time point after transfection. At 72 h post transfection, E-CAD mRNA expression was increased in miR-221-5p overexpressing PC-3M-Pro4luc2 cells (Additional file 7: Figure S6 left panel; *p* < 0.0001 at 72 h) resulting in increased E-CAD/N-CAD ratio and E-CAD/VIM ratio compared to control (Fig. 5g; *p* < 0.0001 at 72 h for both ratios). 2 weeks post transfection, the expression of EMT markers displayed a different pattern compared to early time point (Additional file 7: Figure S6 right panel) and the E-CAD/N-CAD ratio and E-CAD/VIM ratio had significantly decreased to similar levels as the control (Fig. 5g; *p* < 0.0001 for both ratios).

These data demonstrate that the observed functional and transcriptional changes correlate with miR-221-5p levels.

## Discussion

In this in vitro and in vivo preclinical study, we demonstrated that miR-221-5p acts as tumor suppressor in PCa. miR-221 is generally overexpressed in epithelial cancers and plays an oncogenic role [21]. However, in prostate cancer, oncogenic [25, 34, 35, 44, 58] and tumor suppressive function for miR-221 have been described [27, 28, 41, 42]. Here we reported downregulation of miR-221-5p in PCa cell lines compared to non-cancerous prostatic epithelial Ep156T cells implying an important role for miR-221-5p in the maintenance of a normal epithelial phenotype. Proliferation was decreased by miR-221-5p overexpression in PC-3M-Pro4luc2 cells and C4–2 cells. Notably, we observed faster cell cycle progression in PC-3M-Pro4luc2 cells than in C4–2 cells despite higher baseline expression of miR-221-5p in PC-3M-Pro4luc2 cells. The high proliferation rate of PC-3M-Pro4luc2 cells can be attributed to their high expression of TGF-α and EGF-R as well as p53 mutation and PTEN deficiency which might compensate for intrinsically higher miR-221-5p levels [59]. However, miR-221-5p overexpression significantly reduced proliferation of PC-3M-Pro4luc2 cells. Knock-down of miR-221-5p had no functional effects on PC-3M-Pro4luc2 cells. Despite specific action of anti-miR-221-5p, we achieved a miR-221-5p downregulation of 40%. Given the redundant action of miRNAs and the endogenously low miR-221-5p expression levels in PCa cell lines compared to normal prostatic epithelial cells, the in vitro miR-221-5p downregulation might be too weak to see a positive functional effect. Previously published work demonstrated miR-221-5p overexpression to reduce proliferation of PCa cells by downregulation of cell cycle regulatory proteins [27]. However, miR-221-5p overexpression has also been reported to promote proliferation of PCa cell lines by activating Ras/Raf/MEK/ERK pathway and consequently stable silencing of miR-221-5p reduced tumor growth of PCa cell xenografts [44]. These studies imply that miR-221-5p targets a multitude of pathways and acts as tumor suppressor or oncogenic miRNA depending on the cellular and experimental conditions. For a better understanding of miR-221-5p function during PCa growth, we studied miR-221-5p in a transient overexpression orthotopic in vivo model. miR-221-5p overexpression significantly reduced tumor growth and tumor volume compared to control. Tumors did not display differences in proliferation or apoptosis rates as determined by PCNA and cl. casp-3 staining, which is likely due to the loss of overexpressed miR-221-5p in the course of the in vivo experiment. Indeed, miR-221-5p diminished significantly over 2 weeks after overexpression in vitro, which was associated with loss of effect on proliferation. This supports the hypothesis that the observed tumor suppressive effects are dependent on miR-221-5p (over)expression levels and might also account for the lack of detectable direct effects of miR-221-5p overexpression in the dissected tumors. While there is still a significant difference in tumor growth in vivo after 28 days, the effect of miR-221-5p overexpression on cell growth was lost after 12 days in vitro (Fig. 5c). This is due to the fact that control cells reached confluency earlier than miR-221-5p overexpressing cells and were not proliferating anymore. In contrast, scrambled transfected cells proliferated at a high rate at 2 weeks post injection in vivo and did not experience any spatial limitations for growth. Despite transient transfection, miR-221-5p overexpression reduced tumor burden significantly in vivo suggesting an initially large inhibitory effect of miR-221-5p overexpression on PCa growth. Our data imply that transient miR-221-5p overexpression reduces PCa cell proliferation in vitro and influences the growth kinetic of orthotropic tumors in vivo. Analysis of publicly available microRNA dataset (GSE21036) [45] led us to the finding that miR-221-5p expression is progressively decreased in PCa tumor and further decreases in metastasis. Low miR-221-5p expression correlated with lower histological differentiation as assessed by Gleason score and with disease progression as assessed by TNM staging, indicating that miR-221-5p downregulation is a pre-requisite for local and advanced disease in PCa patients. The significance of miR-221 as tumor suppressor in PCa is supported by the notion, that miR-221 is downregulated in clinical specimens of TMPRMSS2:ERG fusion positive PCa, which comprise over 50% of all diagnosed tumors [60]. Moreover, low miR-221 levels in PCa tissue are associated with earlier recurrence after radical prostatectomy [61].

The ability to undergo unlimited division and to form clones is an essential feature of aggressive cancer cells. miR-221-5p overexpression significantly reduced colony formation suggesting that miR-221-5p might interfere with self-renewal mechanisms. Interestingly, murine miR-221-5p has been shown to directly target and downregulate Oct4, Nanog, Sox2, Klf4 and PRMT7 in mouse embryonic stem cells thereby functioning as “anti-stemness” miRNA [62]. In human PCa cells, miR-221-3p has been shown to target the stem cell factor Bmi-1 [41]. Oct4, Nanog, Sox2 and Bmi-1 are upregulated in PCa tissue and tumor-initiating PCa cells [63, 64]. However, additional studies are required to elucidate the mechanistic effect of miR-221 and its interaction with transcription factors implicated in self-renewal and cell dedifferentiation, leading to highly aggressive, metastatic disease.

Epithelial-to-mesenchymal transition (EMT) is an established characteristic of highly aggressive cancer cells. Differential expression of E-CAD, N-CAD or VIM upon miR-221-5p overexpression has already been reported in PCa cell lines [27, 44]. We showed that miR-221-5p overexpression induced an increase of the E-CAD/VIM ratio on mRNA and protein level in PC-3M-Pro4luc2 cells leading to a more epithelial phenotype, which can be mainly attributed to a decrease of VIM protein expression. In contrast to the highly mesenchymal PC-3M-Pro4luc2 cells, C4–2 cells express intrinsically lower VIM levels, which are not affected by miR-221-5p overexpression leading to a decreased E-CAD/VIM ratio in miR-221-5p overexpressing C4–2 cells. Our data support the notion that the EMT expression profile of PC-3M-Pro4luc2 cells and C4–2 cells are differentially affected by miR-221-5p overexpression. Additionally, miR-221-5p overexpression reduced migration of both cell lines dramatically. PC-3M-Pro4luc2 cells display elongated morphology and migrate in a “mesenchymal manner” [65]. In contrast, C4–2 cells show a less mesenchymal phenotype and migrate in an “amoeboid manner” [65, 66]. We have previously shown that different molecular mechanisms support motility in PC-3M-Pro4luc2 and C4–2B cells [51]. Interestingly, with the diminished miR-221-5p overexpression levels 2 weeks post transfection the migration of PC-3 M-Pro4luc2 cells was significantly increased compared to control (Fig. 5f). It has previously been shown for miR-430 that miRNAs can balance the expression of agonist/antagonist pairs [67]. Similarly, the moderately increased miR-221-5p levels at 2 weeks post transfection in combination with cellular compensatory mechanisms [12, 68] might have led to an imbalance between positive and negative regulators of migration resulting in increased migration. The ability to migrate is a pre-requisite for tumor cell extravasation and metastasis formation. This highly dynamic process requires engagement of adhesion molecules including selectins, cadherins, integrins, CD44 and immunoglobulin superfamily receptors [69]. In line with the modulation of E-CAD and VIM expression and reduced migration in vitro by miR-221-5p overexpression, extravasation of highly aggressive PC-3M-Pro4_mCherry cells was reduced by miR-221-5p in vivo, in a zebrafish model of tumor cell extravasation. miR-221-5p transfected PC-3M-Pro4_mCherry cells formed less and smaller metastatic foci at the caudal hematopoietic tissue (CHT) of zebrafish embryos, suggesting a critical role of miR-221-5p to prevent extravasation at distant sites. These results matched our in vitro observations and similarly as in the mouse experiment, miR-221-5p overexpression reduced tumor burden in zebrafish embryos.

Taken together, our findings and the loss of miR-221 during PCa progression [27, 33], might suggest a role for miR-221 in counteracting tumor cell migration and metastasis formation. Accordingly, we found lower miR-221-5p levels in the more advanced, androgen-independent C4–2 cells compared to the parental, androgen-dependent LNCaP cell line [50]. VCaP cells were derived from a vertebral metastasis and represent a model of advanced, metastatic PCa. miR-221-5p levels were nearly undetectable in VCaP cells. This finding aligns with downregulation of miR-221-5p in metastatic disease compared to primary tumor as we found by analysis of a publicly available patient dataset. Increased methylation of the miR-221/− 222 locus and miR-221-5p suppression has been associated with metastatic PCa and might contribute to disease progression [27]. We observed higher miR-221-5p expression in AR^−^ cell lines than in AR^+^ PCa cell lines, which is possibly due to the absence of AR, a transcriptional repressor of the miR-221/− 222 gene cluster [70]. ADT resistance and progression to metastasis is often linked to restored AR activity [71], which potentially leads to repression of miR-221/− 222 in CRPC and could further promote PCa progression in this critical phase. However, additional experiments are necessary to elucidate the relation between miR-221-5p and AR status.

## Conclusion

Dataset analysis revealed downregulation of miR-221 (3p and 5p) during PCa progression and suggests a tumor suppressive role of miR-221 in PCa patients. We report that miR-221-5p acts as tumor suppressor miRNA in PCa cell line models and reduces tumor burden in mouse and zebrafish in vivo models. miR-221 was identified as key regulator of a network of other miRNAs in PCa [31] and has the potential to drastically modulate cell physiology. Therefore, a better understanding of miR-221 function in the context of PCa will potentially lead to the identification of novel interactions between signalling pathways that promote PCa progression.

## Additional files

Additional file 1: **Table S1.** Body weight and tumor size of mouse in vivo experiment. Table S2: Primer sequences. (DOCX 16 kb)

Additional file 2: **Figure S1.** miR-221 is downregulated during PCa progression in patient samples. (a) microRNA dataset analysis in GSE21036 dataset. Expression of miR-221-3p in 113 PCa samples compared to 28 normal tissue samples. Fold change (FC = 0.85) was calculated and data was analysed by t-test. (b) Analysis of miR-221-3p expression in metastatic PCa samples compared to primary PCa tissue in GSE21036 dataset. Fold change (FC = 0.74) was calculated and data analysed by t-test. (c) Data of GSE21036 was grouped according to the indicated Gleason score (GS) and miR-221-3p expression analysed. Adjusted *p*-value was calculated by one-way ANOVA. (d) miR-221-3p expression (GSE21036) was analysed in samples grouped for pathological stage (T). Data was analysed by one-way ANOVA. (e) Analysis of miR-221-5p expression in samples grouped for clinical stage (T) in GSE21036 dataset. Data was analysed by one-way ANOVA. (f) Analysis of miR-221-3p expression in sample groups according to clinical stage (T). Data of GSE21036 was analysed by one-way ANOVA. (PDF 280 kb)

Additional file 3: **Figure S2.** miR-221-5p exerts tumor suppressive function on PCa cell lines in vitro. (a) Cell cycle of wild-type PC-3 M-Pro4luc2 and C4–2 cells was analysed by PI staining at 0 h, 16 h and 48 h after release starvation. The frequency of cells in G1, S and G2/M phase was quantified by Dean-Jett-Fox model. Results of *n* = 2 technical replicates were analysed by unpaired, two-tailed t-test. * *p* < 0.05, ** *p* < 0.01. (b) PC-3 M-Pro4luc2 cells were transfected with 10 nM miR-221-5p or 10 nM scrambled. The next day, cells were transfected with increasing concentrations of anti-miR-221-5p (10 nM, 20 nM, 30 nM, 60 nM and 120 nM) or 10 nM anti-scrambled. miR-221-5p expression was assessed 48 h later and LOG difference (LOG(2-ΔΔCt)) to scrambled/anti-scrambled co-transfection control calculated. Data of *n* = 3 technical replicates are represented and were analysed by two-way ANOVA with Sidak’s multiple comparison test. **** *p* < 0.0001 miR-221-5p/anti-miR-221-5p compared to scrambled/anti-scrambled control, #### *p* < 0.0001 scrambled/anti-miR-221-5p compared to scrambled/anti-scrambled control, *p* < 0.0001 miR-221-5p/anti-miR-221-5p compared to scrambled/anti-miR-221-5p. (c) miR-221-5p overexpression in C4–2 cells 72 h post transfection with miR-221-5p or scrambled control. LOG difference to scrambled was calculated as LOG(2-ΔΔCt). Data of one representative experiment are shown and were analysed by unpaired, two-tailed t-test. **** *p* < 0.0001. (d) Proliferation of miR-221-5p and scrambled overexpressing C4–2 cells. Images were taken 72 h post transfection. Proliferation was measured by MTS at four time points (0 h, 24 h, 48 h and 72 h). Data of *n* = 3 independent experiments are shown as fold change normalised to T’0 h and were analysed by two-way ANOVA with repeated measures by both factors with Sidak’s multiple comparison test. *** *p* < 0.001, **** *p* < 0.0001. (e) Clonogenicity assay of C4–2 cells transfected with miR-221-5p or scrambled. All technical replicates of three independent experiments (*n* = 3) were pooled and fold change to scrambled was calculated. Data were analysed by unpaired, two-tailed t-test. **** *p* < 0.0001. (PDF 245 kb)

Additional file 4: **Figure S3.** miR-221-5p overexpression affects plasticity of PCa cells and EMT marker expression. (a) EMT marker expression in PC-3 M-Pro4luc2 cell overexpressing miR-221-5p or negative scrambled control. Data of three independent experiments (*n* = 3) are shown as LOG difference to control (LOG(2^-ΔΔCt^)) and were analysed by paired, two-tailed t-test. * *p* < 0.05, ** *p* < 0.01. (b) EMT marker expression in miR-221-5p or scrambled overexpressing C4–2 cells. Results of three independent experiment (*n* = 3) are shown as LOG difference to control (LOG(2^-ΔΔCt^)) and were analysed by paired, two-tailed t-test. * *p* < 0.05; n.d. = not detectable. (c) E-CAD/VIM mRNA ratio of miR-221-5p and scrambled overexpressing C4–2 cells was calculated from relative expression (2^-ΔCt^) and is shown as fold change to scrambled. Results of three independent experiments are shown (*n* = 3). Data were analysed by paired, two-tailed t-test. * *p* < 0.05. (d) E-CAD and VIM protein expression in PC-3 M-Pro4luc2 cells 72 h post transfection with miR-221-5p or scrambled. Protein expression was normalised to β-actin and fold change to scrambled was calculated for *n* = 2 independent experiments. Data were analysed by paired, two-tailed t-test. (e) E-CAD and VIM protein expression in miR-221-5p or scrambled overexpressing C4–2 cells 72 h post transfection. Protein expression was normalised to housekeeping β-actin and fold change to scrambled negative control calculated. Data of *n* = 2 technical replicates are shown and were analysed by unpaired, two-tailed t-test. (f) Western blot for E-CAD and VIM in C4–2 cells overexpressing miR-221-5p or scrambled. Bands were quantified and normalised to housekeeping protein β-actin and E-CAD/VIM ratio was calculated. *N* = 2 technical replicates are shown as fold change to scrambled and were analysed by unpaired, two-tailed t-test. * *p* < 0.05. (g) Migration of miR-221-5p or scrambled overexpressing C4–2 cells. All technical replicates of three independent experiments (*n* = 3) are shown as fold change to scrambled. Data were analysed by unpaired, two-tailed t-test. **** *p* < 0.0001. (PDF 215 kb)

Additional file 5: **Figure S4.** Orthotopic tumor growth is reduced by miR-221-5p overexpression in vivo. (a) miR-221-5p expression in PC-3 M-Pro4luc2 cells 48 h post transfection, prior to intraprostatic injection in mice. Data are shown as LOG difference (LOG(2^-ΔΔCt^)) to scrambled and were analysed by unpaired, two-tailed t-test. **** *p* < 0.0001. (b) Clonogenicity assay of miR-221-5p overexpressing PC-3 M-Pro4luc2 cells. Same batch of cells was used for in vivo inoculation. Data were analysed by unpaired, two-tailed t-test. (c) BLI images of all mice are shown at 7 and 28 days post implantation. Mice without detectable BLI signal (M4 and M10) at the end of the experiment were omitted from further analysis. (d) Tumors dissected from mice injected with miR-221-5p or scrambled transfected PC-3 M-Pro4luc2 cells at the end of the experiment. Images and tumor volume of all mice are shown. Scale bar = 5 mm. (PDF 352 kb)

Additional file 6: **Figure S5.** Morphology of orthotopically grown PC-3 M-Pro4luc2 tumors is not affected by miR-221-5p overexpression. H&E and immunofluorescence imaging of all dissected tumors. The expression of proliferation marker PCNA (green), apoptosis marker cl. casp-3 (red), tumor cell marker panCK (red) and stroma marker α-SMA (green) was analysed by immunofluorescence staining. Representative images of each mouse are shown. (PDF 988 kb)

Additional file 7: **Figure S6.** Differential EMT marker expression at early and late time point post transfection. Expression of selected EMT markers in PC-3 M-Pro4luc2 cells at 72 h and 2 weeks post transfection. LOG difference was calculated as LOG(2^-ΔΔCt^) normalised to scrambled. Results of *n* = 3 technical triplicates are shown and data were analysed by unpaired, two-tailed t-test. * *p* < 0.05, ** *p* < 0.01, **** *p* < 0.0001. (PDF 35 kb)

## Data Availability

All data generated or analysed during this study are included in this published article [and its supplementary information files].

## Abbreviations

ADT: Androgen deprivation therapy
AR: Androgen receptor
CHT: Caudal hematopoietic tissue
cl. casp-3: Cleaved caspase-3
CRPC: Castration-resistant prostate cancer
DHT: Dihydrotestosterone
E-CAD: E-cadherin
EGF: Epidermal growth factor
EMT: Epithelial-mesenchymal transition
miRNAs: microRNAs
N-CAD: N-cadherin
panCK: Pan-cytokeratin
PCa: Prostate cancer
PCNA: Proliferating cell nuclear antigen
RP: Radical prostatectomy
VIM: Vimentin
ZEB-2: Zinc finger E-box-binding homeobox 2
α-SMA: α-smooth muscle actin
