# Supplementary material for: miR-221-5p regulates proliferation and migration in human prostate cancer cells and reduces tumor growth in vivo
Source: BMC Cancer. 2019 Jun 25;19:627. doi: 10.1186/s12885-019-5819-6 (PMC6593572; doi:10.1186/s12885-019-5819-6)
Supplement: Supplementary file 1 — Table S1. Body weight and tumor size of mouse in vivo experiment. Table S2: Primer sequences. (DOCX 16 kb) [file 12885_2019_5819_MOESM1_ESM.docx]

*Table S1: Body weight and tumor size of mouse in vivo experiment.*

|  | **Mouse** | **Body weight** | **Tumor length [mm]** | **Tumor width [mm]** | **Tumor volume [mm^3^]** |
| --- | --- | --- | --- | --- | --- |
| **scrambled** | *M1* | 22g | 7.29 | 6.35 | 153.9 |
|  | *M2* | 23g | 5.27 | 4.46 | 54.9 |
|  | *M3* | 20g | 5.39 | 5.01 | 70.8 |
|  | *M5* | 24g | 6.36 | 6.04 | 121.5 |
| **miR-221-5p** | *M6* | 24g | 5.68 | 4.42 | 58.1 |
|  | *M7* | 22g | 4.38 | 4.25 | 41.4 |
|  | *M8* | 24g | 4.35 | 3.15 | 22.6 |
|  | *M9* | 23g | 4.79 | 4.68 | 54.9 |

*Table S2: Primer sequences.*

| E-cadherin FW | 5’-TTGACGCCGAGAGCTACAC-3’ |
| --- | --- |
| E-cadherin RV | 5’-GACCGGTGCAATCTTCAAA-3’ |
| N-cadherin FW | 5’-CAGACCGACCCAAACAGCAAC-3’ |
| N-cadherin RV | 5’-GCAGCAACAGTAAGGACAAACATC-3’ |
| Vimentin FW | 5’-CCAAACTTTTCCTCCCTGAACC-3’ |
| Vimentin RV | 5’-CGTGATGCTGAGAAGTTTCGTTGA-3’ |
| ZEB-2 FW | 5’-GACCTGGCAGTGAAGGAAAA-3’ |
| ZEB-2 RV | 5’-GGCACTTGCAGAAACACAGA-3’ |
| SNAIL-1 FW | 5’-ACCACTATGCCGCGCTCTT-3’ |
| SNAIL-1 RV | 5’-GGTCGTAGGGCTGCTGGAA-3’ |
| SNAIL-2 FW | 5’-TGTGTGGACTACCGCTGC-3’ |
| SNAIL-2 RV | 5’-TCCGGAAAGAGGAGAGAGG-3’ |
| TWIST FW | 5’-GCCGGAGACCTAGATGTCATT-3’ |
| TWIST RV | 5’-TTTTAAAAGTGCGCCCCACG-3’ |
| HPRT FW | 5’-AGACTTTGCTTTCCTTGGTCAGG-3’ |
| HPRT RV | 5’-GTCTGGCTTATATCCAACACTTCG-3’ |
| β-ACTIN FW | 5’-GAAACTACCTTCAACTCCATC-3’ |
| β-ACTIN RV | 5’-CTAGAAGCATTTGCGGTGGAC-3’ |
