# Supplementary figures and images for: miR-221-5p regulates proliferation and migration in human prostate cancer cells and reduces tumor growth in vivo
Source: BMC Cancer. 2019 Jun 25;19:627. doi: 10.1186/s12885-019-5819-6 (PMC6593572; doi:10.1186/s12885-019-5819-6)

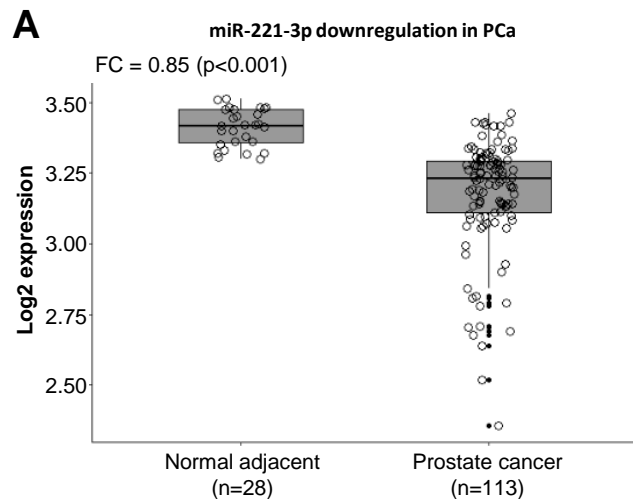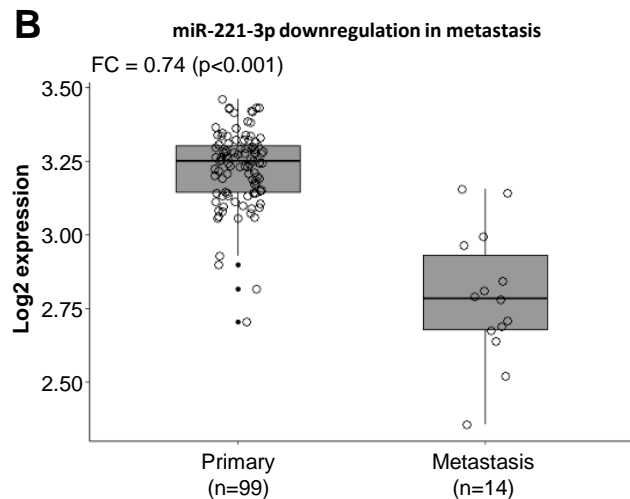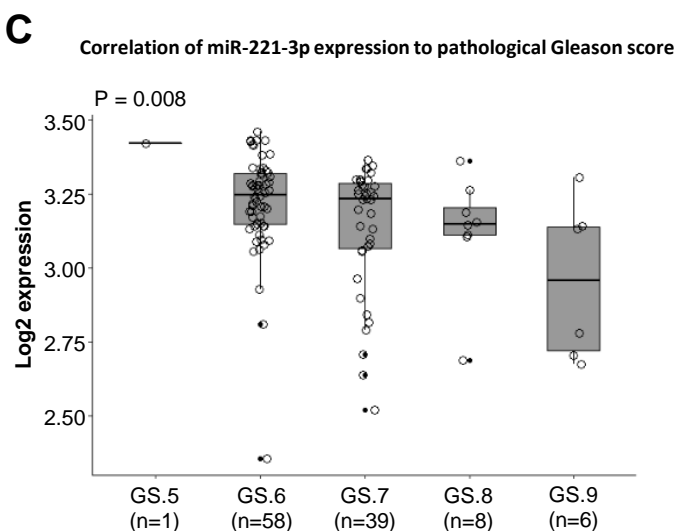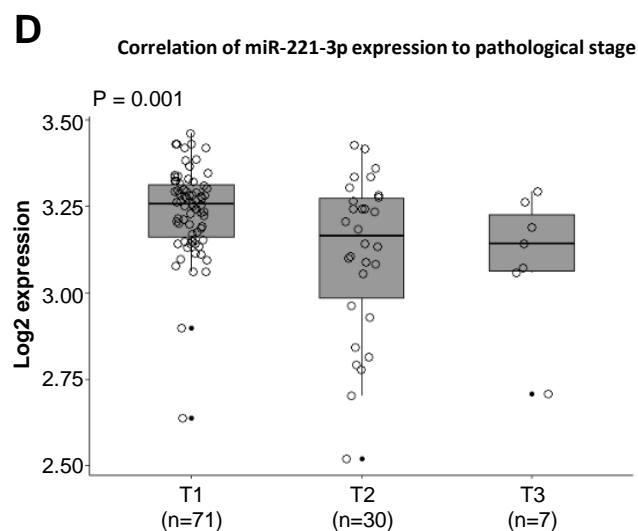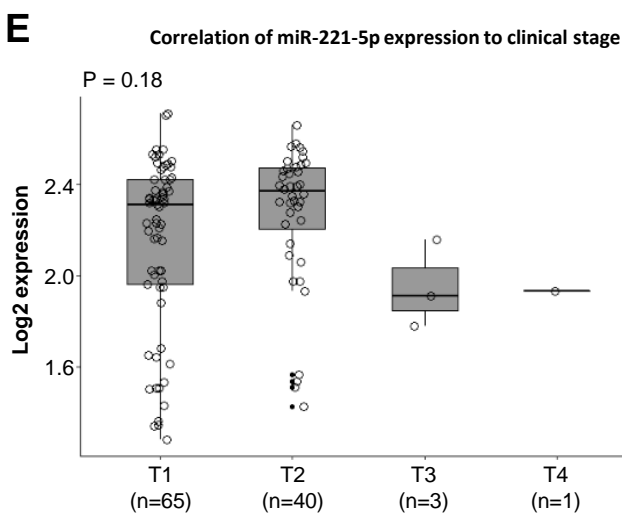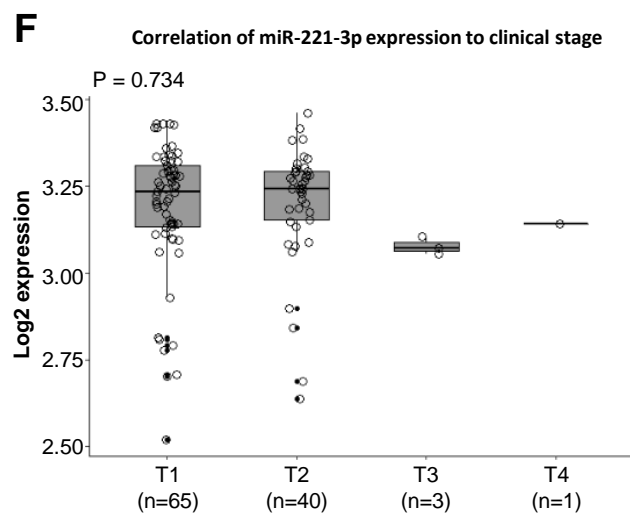

Supplement: Supplementary file 2 — Figure S1. miR-221 is downregulated during PCa progression in patient samples. (a) microRNA dataset analysis in GSE21036 dataset. Expression of miR-221-3p in 113 PCa samples compared to 28 normal tissue samples. Fold change (FC = 0.85) was calculated and data was analysed by t-test. (b) Analysis of miR-221-3p expression in metastatic PCa samples compared to primary PCa tissue in GSE21036 dataset. Fold change (FC = 0.74) was calculated and data analysed by t-test. (c) Data of GSE21036 was grouped according to the indicated Gleason score (GS) and miR-221-3p expression analysed. Adjusted p-value was calculated by one-way ANOVA. (d) miR-221-3p expression (GSE21036) was analysed in samples grouped for pathological stage (T). Data was analysed by one-way ANOVA. (e) Analysis of miR-221-5p expression in samples grouped for clinical stage (T) in GSE21036 dataset. Data was analysed by one-way ANOVA. (f) Analysis of miR-221-3p expression in sample groups according to clinical stage (T). Data of GSE21036 was analysed by one-way ANOVA. (PDF 280 kb) [file 12885_2019_5819_MOESM2_ESM.pdf]

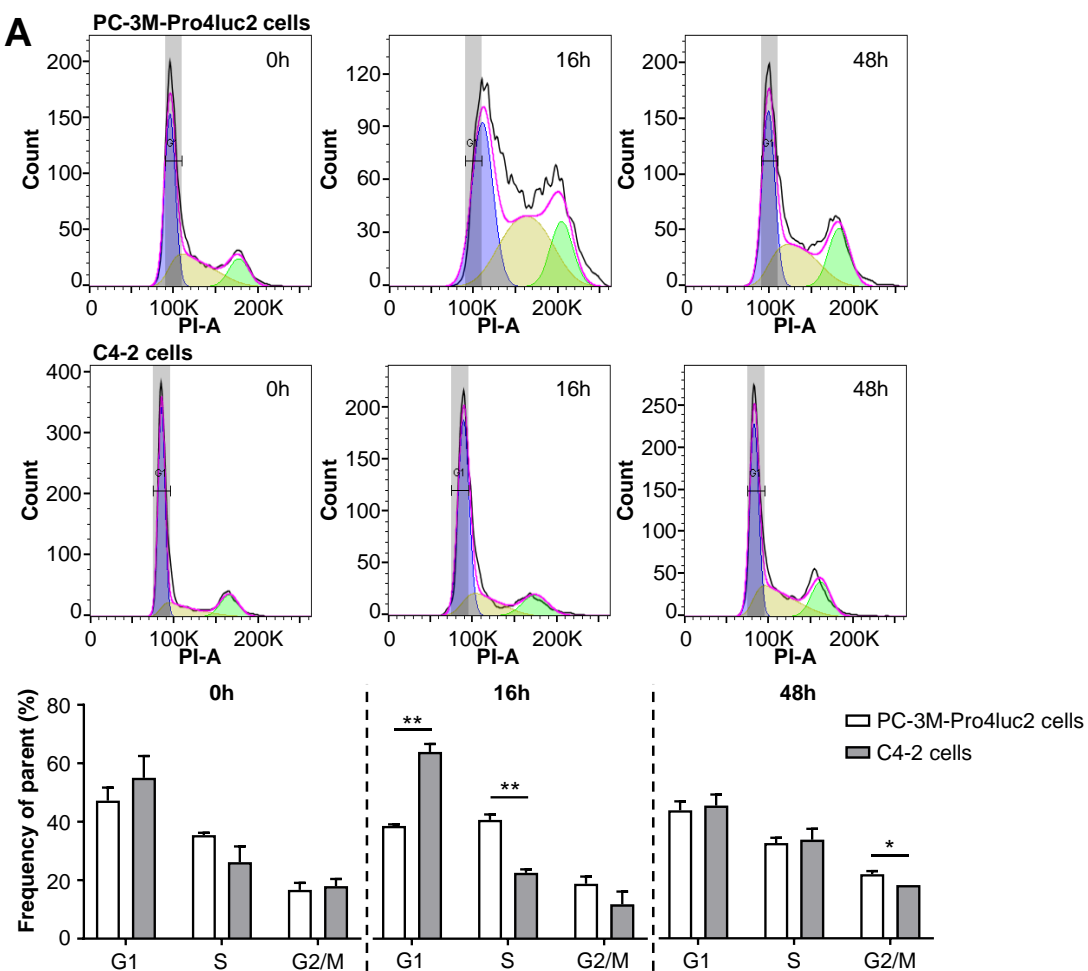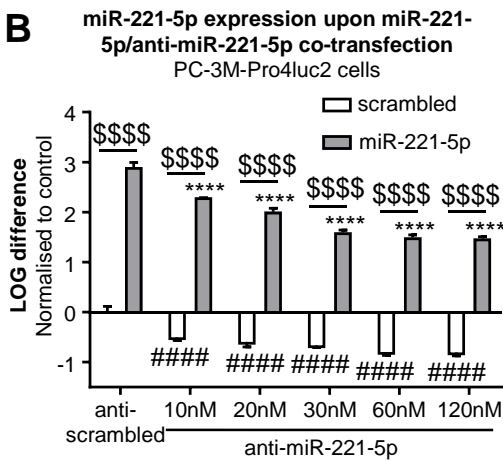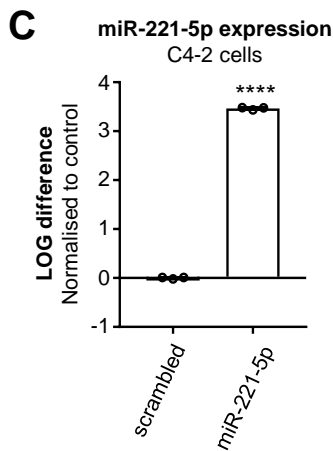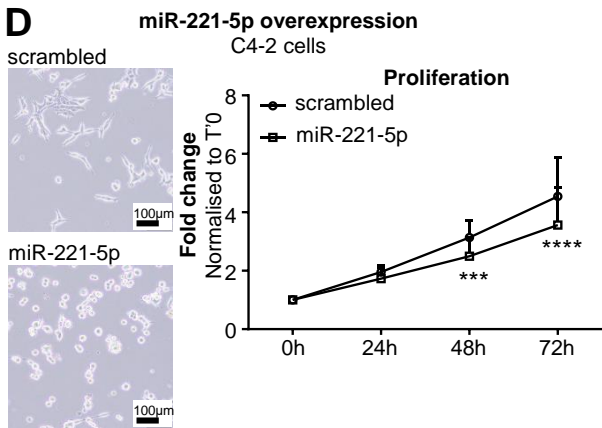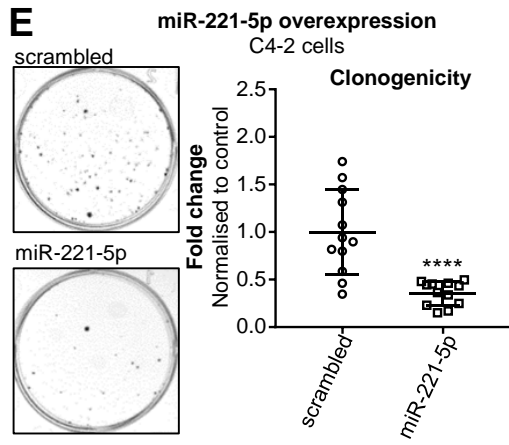

Supplement: Supplementary file 3 — Figure S2. miR-221-5p exerts tumor suppressive function on PCa cell lines in vitro. (a) Cell cycle of wild-type PC-3 M-Pro4luc2 and C4–2 cells was analysed by PI staining at 0 h, 16 h and 48 h after release starvation. The frequency of cells in G1, S and G2/M phase was quantified by Dean-Jett-Fox model. Results of n = 2 technical replicates were analysed by unpaired, two-tailed t-test. * p < 0.05, ** p < 0.01. (b) PC-3 M-Pro4luc2 cells were transfected with 10 nM miR-221-5p or 10 nM scrambled. The next day, cells were transfected with increasing concentrations of anti-miR-221-5p (10 nM, 20 nM, 30 nM, 60 nM and 120 nM) or 10 nM anti-scrambled. miR-221-5p expression was assessed 48 h later and LOG difference (LOG(2-ΔΔCt)) to scrambled/anti-scrambled co-transfection control calculated. Data of n = 3 technical replicates are represented and were analysed by two-way ANOVA with Sidak’s multiple comparison test. **** p < 0.0001 miR-221-5p/anti-miR-221-5p compared to scrambled/anti-scrambled control, #### p < 0.0001 scrambled/anti-miR-221-5p compared to scrambled/anti-scrambled control, p < 0.0001 miR-221-5p/anti-miR-221-5p compared to scrambled/anti-miR-221-5p. (c) miR-221-5p overexpression in C4–2 cells 72 h post transfection with miR-221-5p or scrambled control. LOG difference to scrambled was calculated as LOG(2-ΔΔCt). Data of one representative experiment are shown and were analysed by unpaired, two-tailed t-test. **** p < 0.0001. (d) Proliferation of miR-221-5p and scrambled overexpressing C4–2 cells. Images were taken 72 h post transfection. Proliferation was measured by MTS at four time points (0 h, 24 h, 48 h and 72 h). Data of n = 3 independent experiments are shown as fold change normalised to T’0 h and were analysed by two-way ANOVA with repeated measures by both factors with Sidak’s multiple comparison test. *** p < 0.001, **** p < 0.0001. (e) Clonogenicity assay of C4–2 cells transfected with miR-221-5p or scrambled. All technical replicates of three independe [file 12885_2019_5819_MOESM3_ESM.pdf]

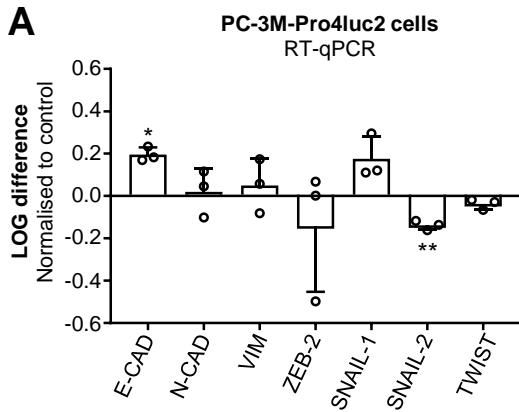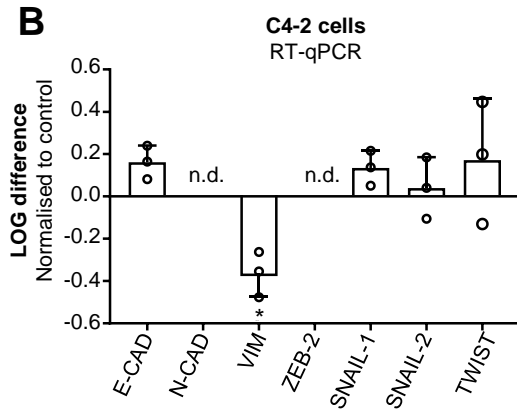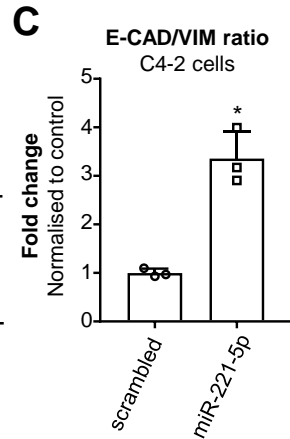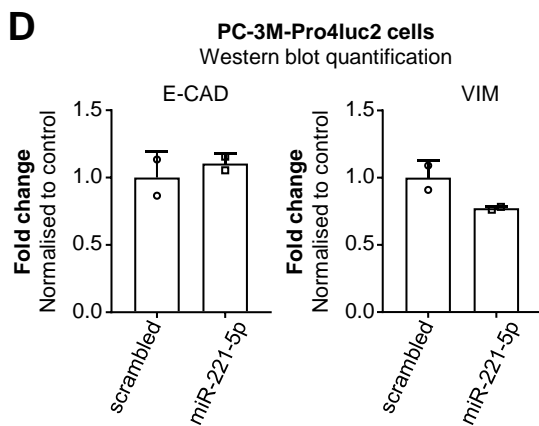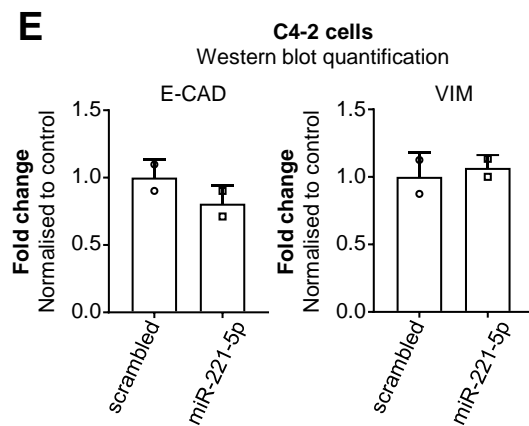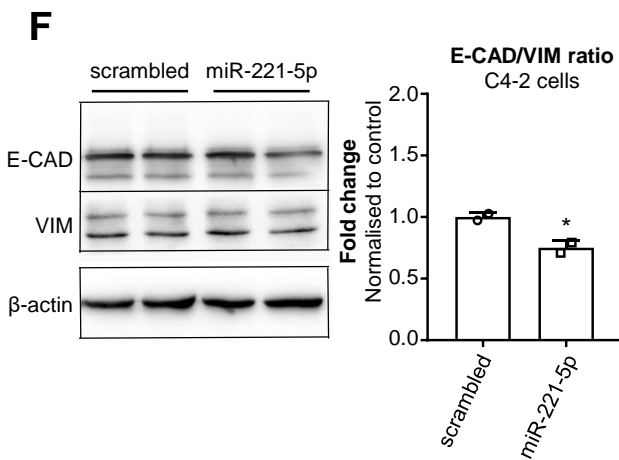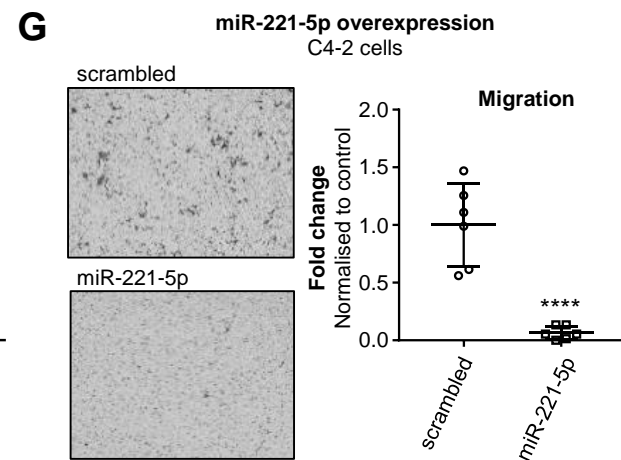

Supplement: Supplementary file 4 — Figure S3. miR-221-5p overexpression affects plasticity of PCa cells and EMT marker expression. (a) EMT marker expression in PC-3 M-Pro4luc2 cell overexpressing miR-221-5p or negative scrambled control. Data of three independent experiments (n = 3) are shown as LOG difference to control (LOG(2-ΔΔCt)) and were analysed by paired, two-tailed t-test. * p < 0.05, ** p < 0.01. (b) EMT marker expression in miR-221-5p or scrambled overexpressing C4–2 cells. Results of three independent experiment (n = 3) are shown as LOG difference to control (LOG(2-ΔΔCt)) and were analysed by paired, two-tailed t-test. * p < 0.05; n.d. = not detectable. (c) E-CAD/VIM mRNA ratio of miR-221-5p and scrambled overexpressing C4–2 cells was calculated from relative expression (2-ΔCt) and is shown as fold change to scrambled. Results of three independent experiments are shown (n = 3). Data were analysed by paired, two-tailed t-test. * p < 0.05. (d) E-CAD and VIM protein expression in PC-3 M-Pro4luc2 cells 72 h post transfection with miR-221-5p or scrambled. Protein expression was normalised to β-actin and fold change to scrambled was calculated for n = 2 independent experiments. Data were analysed by paired, two-tailed t-test. (e) E-CAD and VIM protein expression in miR-221-5p or scrambled overexpressing C4–2 cells 72 h post transfection. Protein expression was normalised to housekeeping β-actin and fold change to scrambled negative control calculated. Data of n = 2 technical replicates are shown and were analysed by unpaired, two-tailed t-test. (f) Western blot for E-CAD and VIM in C4–2 cells overexpressing miR-221-5p or scrambled. Bands were quantified and normalised to housekeeping protein β-actin and E-CAD/VIM ratio was calculated. N = 2 technical replicates are shown as fold change to scrambled and were analysed by unpaired, two-tailed t-test. * p < 0.05. (g) Migration of miR-221-5p or scrambled overexpressing C4–2 cells. All technical replicates of three independent experiments (n = 3) are [file 12885_2019_5819_MOESM4_ESM.pdf]

**A** miR-221-5p expression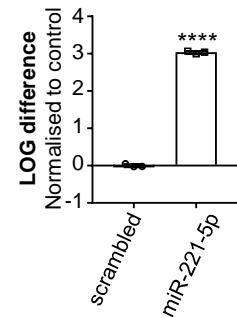**B** Clonogenicity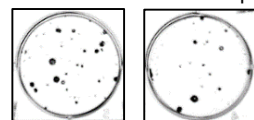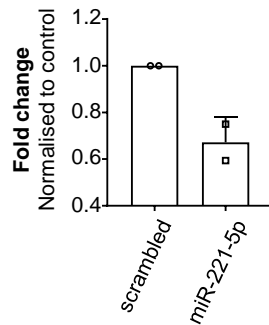**C**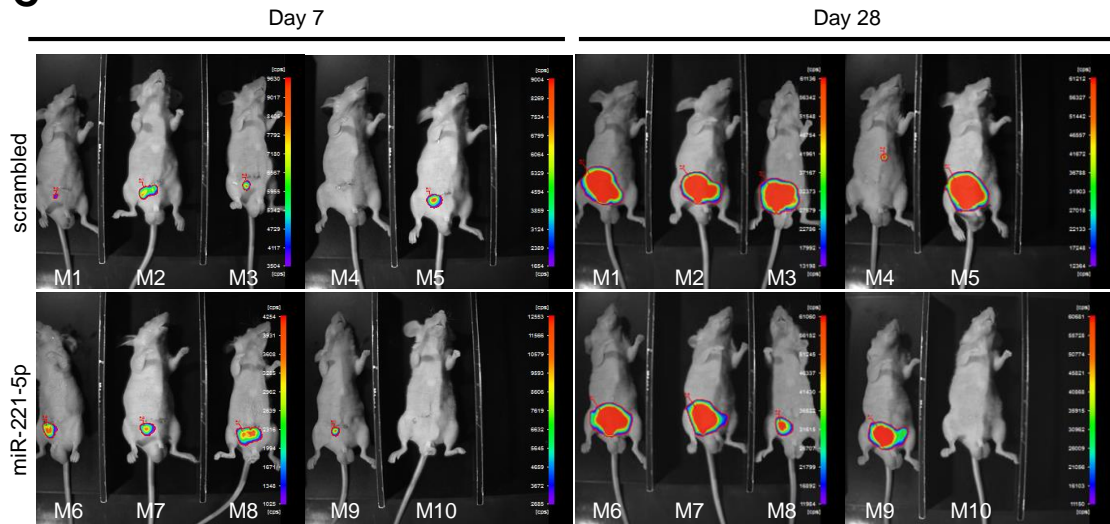**D**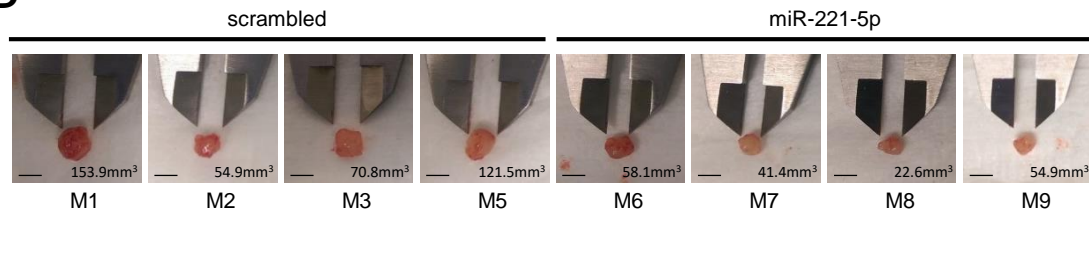

Supplement: Supplementary file 5 — Figure S4. Orthotopic tumor growth is reduced by miR-221-5p overexpression in vivo. (a) miR-221-5p expression in PC-3 M-Pro4luc2 cells 48 h post transfection, prior to intraprostatic injection in mice. Data are shown as LOG difference (LOG(2-ΔΔCt)) to scrambled and were analysed by unpaired, two-tailed t-test. **** p < 0.0001. (b) Clonogenicity assay of miR-221-5p overexpressing PC-3 M-Pro4luc2 cells. Same batch of cells was used for in vivo inoculation. Data were analysed by unpaired, two-tailed t-test. (c) BLI images of all mice are shown at 7 and 28 days post implantation. Mice without detectable BLI signal (M4 and M10) at the end of the experiment were omitted from further analysis. (d) Tumors dissected from mice injected with miR-221-5p or scrambled transfected PC-3 M-Pro4luc2 cells at the end of the experiment. Images and tumor volume of all mice are shown. Scale bar = 5 mm. (PDF 352 kb) [file 12885_2019_5819_MOESM5_ESM.pdf]

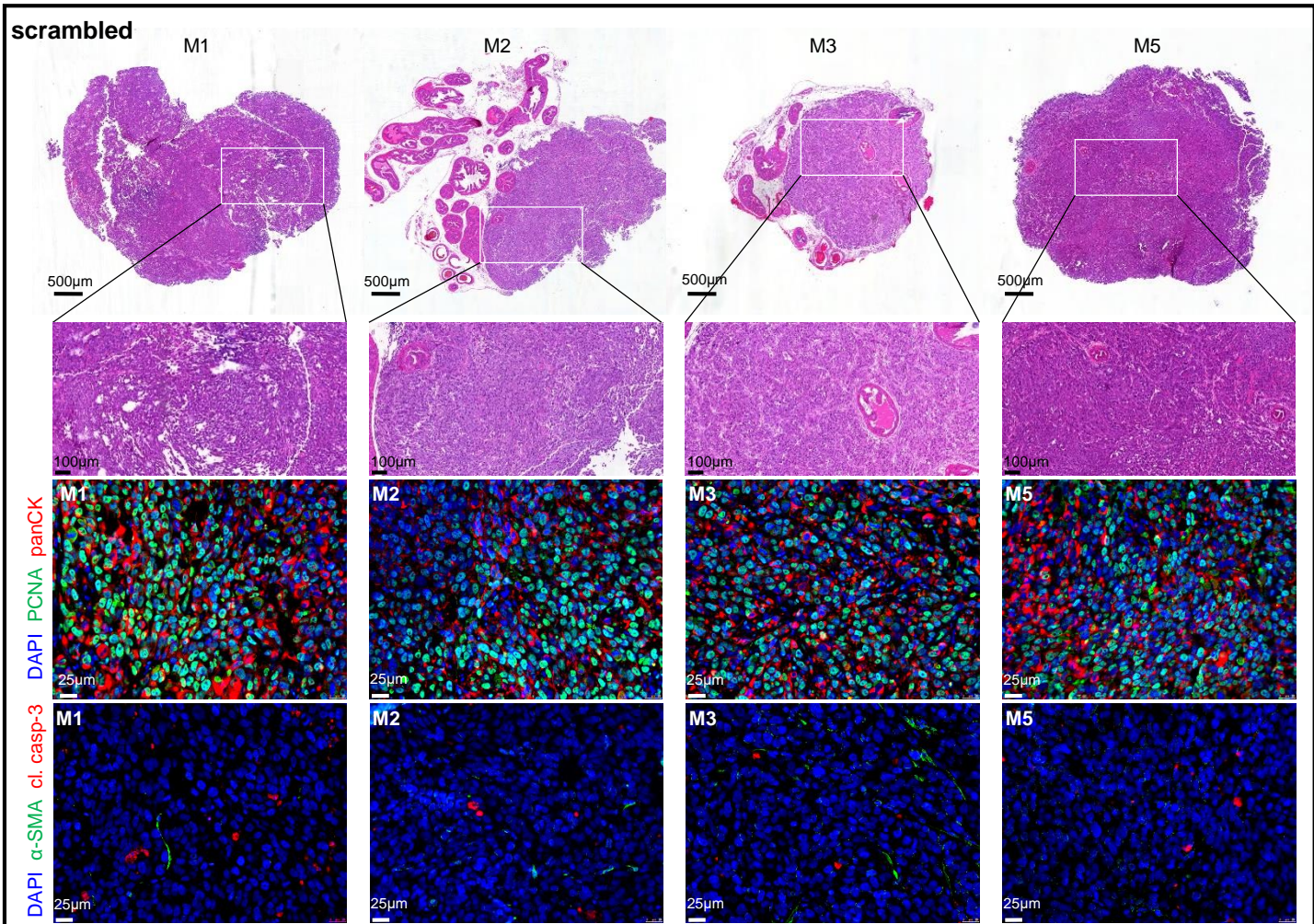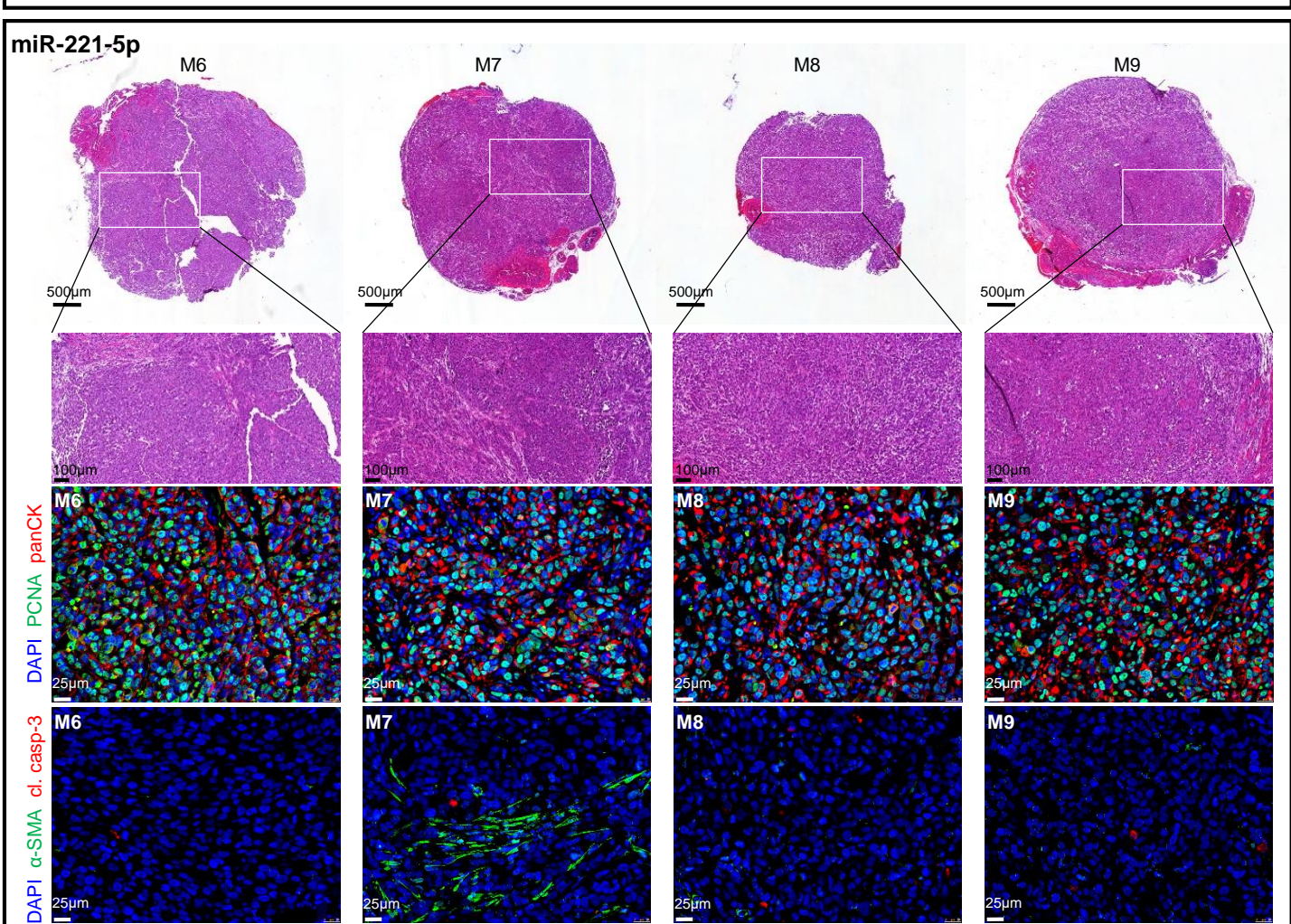

Supplement: Supplementary file 6 — Figure S5. Morphology of orthotopically grown PC-3 M-Pro4luc2 tumors is not affected by miR-221-5p overexpression. H&E and immunofluorescence imaging of all dissected tumors. The expression of proliferation marker PCNA (green), apoptosis marker cl. casp-3 (red), tumor cell marker panCK (red) and stroma marker α-SMA (green) was analysed by immunofluorescence staining. Representative images of each mouse are shown. (PDF 988 kb) [file 12885_2019_5819_MOESM6_ESM.pdf]

72h

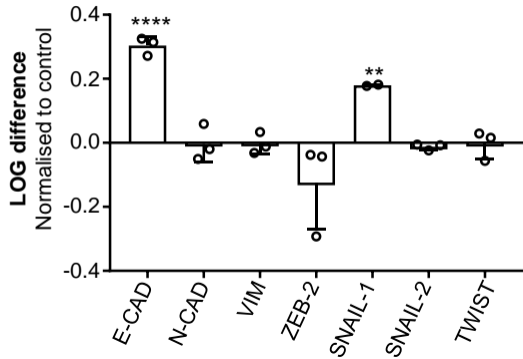

2 weeks

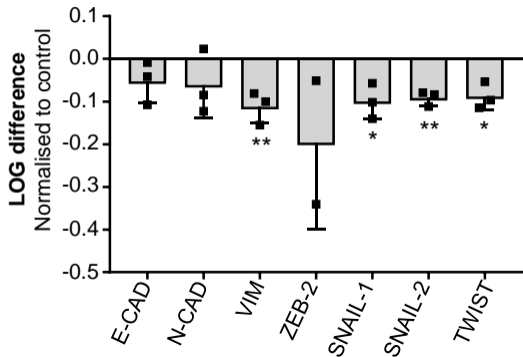

Supplement: Supplementary file 7 — Figure S6. Differential EMT marker expression at early and late time point post transfection. Expression of selected EMT markers in PC-3 M-Pro4luc2 cells at 72 h and 2 weeks post transfection. LOG difference was calculated as LOG(2-ΔΔCt) normalised to scrambled. Results of n = 3 technical triplicates are shown and data were analysed by unpaired, two-tailed t-test. * p < 0.05, ** p < 0.01, **** p < 0.0001. (PDF 35 kb) [file 12885_2019_5819_MOESM7_ESM.pdf]
